# Divergent evolution of genetic sex determination mechanisms along environmental gradients

**DOI:** 10.1093/evlett/qrad011

**Published:** 2023-04-01

**Authors:** Martijn A Schenkel, Jean-Christophe Billeter, Leo W Beukeboom, Ido Pen

**Affiliations:** Groningen Institute for Evolutionary Life Sciences, University of Groningen, Groningen, The Netherlands; Groningen Institute for Evolutionary Life Sciences, University of Groningen, Groningen, The Netherlands; Groningen Institute for Evolutionary Life Sciences, University of Groningen, Groningen, The Netherlands; Groningen Institute for Evolutionary Life Sciences, University of Groningen, Groningen, The Netherlands

**Keywords:** clinal distribution, environmental sex determination, female determiner, genetic sex determination, polymorphism, housefly, male determiner, sex chromosomes, temperature, *transformer*

## Abstract

Sex determination (SD) is a crucial developmental process, but its molecular underpinnings are very diverse, both between and within species. SD mechanisms have traditionally been categorized as either genetic (GSD) or environmental (ESD), depending on the type of cue that triggers sexual differentiation. However, mixed systems, with both genetic and environmental components, are more prevalent than previously thought. Here, we show theoretically that environmental effects on expression levels of genes within SD regulatory mechanisms can easily trigger within-species evolutionary divergence of SD mechanisms. This may lead to the stable coexistence of multiple SD mechanisms and to spatial variation in the occurrence of different SD mechanisms along environmental gradients. We applied the model to the SD system of the housefly, a global species with world-wide latitudinal clines in the frequencies of different SD systems, and found that it correctly predicted these clines if specific genes in the housefly SD system were assumed to have temperature-dependent expression levels. We conclude that environmental sensitivity of gene regulatory networks may play an important role in diversification of SD mechanisms.

## Introduction

Sex determination (SD) is a crucial aspect of the development of sexually reproducing organisms, yet the regulatory mechanisms underlying SD are very diverse and prone to evolutionary change (Bachtrog et al., 2014; Beukeboom & Perrin, 2014). SD mechanisms have traditionally been classified as either environmental (ESD) or genetic (GSD) depending on the type of signal that initiates the determination of an individual’s sex. Under ESD, such signals include temperature, salinity, and acidity during a sensitive period in embryonic development (reviewed in Devlin & Nagahama, 2002; Janzen & Paukstis, 1991). Under GSD, the signal is a specific gene (or set of genes) present in zygotes, leading to either male or female development, such as the male-determining *Sex-determining Region Y (SRY)* gene in mammals or *transformer* (*tra*) in many insects (Berta et al., 1990; Goodfellow & Lovell-Badge, 1993; Pane et al., 2005; Sinclair et al., 1990; Verhulst et al., 2010). Diversification of SD mechanisms occurs via the evolution of novel SD mechanisms that replace their predecessors, a process called SD transition. SD transitions are prevalent in some taxa but not in others (Bachtrog et al., 2014; Beukeboom & Perrin, 2014), indicating variable evolvability of SD systems. What enhances the evolvability of some SD systems but not others and what causes SD transitions is still poorly understood.

One often-overlooked aspect of the evolution of SD is how environmental and genetic factors may simultaneously affect SD. Rather than being mutually exclusive, ESD and GSD could instead be considered as two extremes of a continuum (Beukeboom & Perrin, 2014; Pen et al., 2010; Uller & Helanterä, 2011), with mixed systems occurring in several organismal groups, such as amphibians, fish, and insects (Devlin & Nagahama, 2002; Doums et al., 1996; Hamm et al., 2015; Ma et al., 2016; Mezzasalma et al., 2021; Nigro et al., 2007; Pen et al., 2010). In such mixed systems, SD may reflect a delicate balance between genetic effects that bias the process of SD toward male or female development, counteracted by environmental effects that push the system in the opposite direction (Alho et al., 2010; Holleley et al., 2015). GSD has repeatedly evolved in species which previously had ESD (e.g., Ezaz et al., 2009), and several theoretical models have been developed to predict when such turnovers should occur (Muralidhar & Veller, 2018; Pen et al., 2010; Van Dooren & Leimar, 2003). However, such models typically do not explicitly consider the underlying molecular mechanisms of sex determination. Although sex is determined by genes in species with GSD, environmental conditions can affect SD by modifying the expression levels of these genes (Hodson et al., 2017; Schmidt et al., 1997). Despite clear evidence that such environmental effects may perturb the action of GSD mechanisms (e.g., Devlin & Nagahama, 2002), their effect on the evolution of GSD systems is still unknown.

In many species, SD involves hierarchical gene regulation (Kopp, 2012; Pomiankowski et al., 2004; Wilkins, 1995), where an initial signal targets a small number of regulatory genes that in turn regulate downstream targets. Evolutionary transitions between GSD systems are thought to occur primarily by the displacement of the initial signal gene by another gene with a similar function, or by the recruitment of a new regulatory gene on top of the ancestral SD regulatory pathway (Bull & Charnov, 1977; Pomiankowski et al., 2004; Wilkins, 1995). Genes downstream of the top regulatory genes are considered to be more constrained in terms of evolutionary change, as mutations in such genes may interfere with their pre-existing function in regulating SD. Nonetheless, they are not fully prohibited from undergoing further evolution, and some changes may still occur (Herpin et al., 2013).

**Figure 1.**
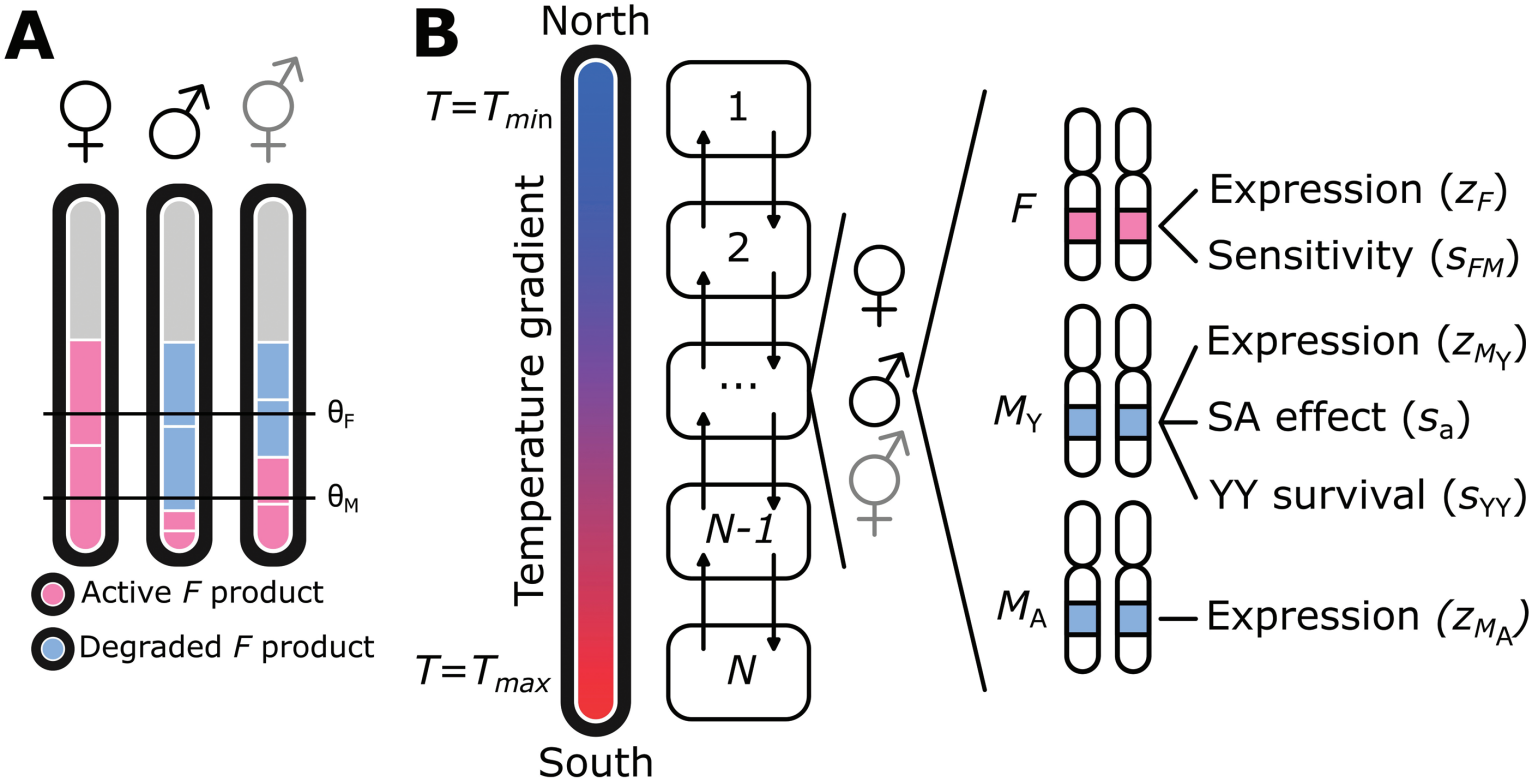
Model overview. (A) Sex is determined based on the net total active *F* product. Active *F* is produced by the *F* locus, and degraded by *M*, produced by *M*_Y_ and/or *M*_A_. Higher temperature increases expression levels of *F*. If the net total active *F* product exceeds a threshold $\theta_{F}$, individuals become females, whereas below the threshold $\theta_{M}$ individuals become males; otherwise, individuals develop into infertile intersexes. (B) Demes 1 through *N* are arranged along a linear gradient where *T* increases from $T_{min}\;$ to $T_{max}$. Each deme contains a variable number of females, males, and intersexes. Reproduction occurs by mating between males and females within the same deme; intersexes do not reproduce. Dispersal occurs at a rate *d* to neighboring demes (indicated by arrows). Every individual has three gene loci *F*, *M*_Y_, and *M*_A_ that jointly determine sex.

A new evolutionary framework needs to integrate the views that (1) SD is not solely environmentally or genetically determined but is to varying degrees affected by both types of cues; and (2) changes in SD cascades do not only occur at the top but may also occur via changes in the underlying genetic network. Here, we formalize this framework by developing a theoretical model of the evolution of SD systems in the presence of spatial environmental variation that affects expression of SD genes. The model is inspired by the polymorphic SD system of the housefly *Musca domestica*, but can be applied more broadly to other systems as well (Table 1). In this system, temperature is likely to act as an environmental cue because (1) variation in SD systems is correlated with variation in temperature between natural populations, and (2) temperature affects SD in several *M. domestica* strains harboring mutant SD genes (see also Box 1). Like in *M. domestica* SD, the model features two types of SD genes: an environmentally-sensitive gene *F* induces femaleness when active, and one or more *M*-genes that induce maleness by inhibiting *F* (Figure 1, see details below). We investigate here how the (co-)evolution of *F* and *M* can yield novel SD systems under the influence of environmental sensitivity of *F*. We then use the model to explain how the multifactorial SD system of houseflies has evolved (Box 1).

**Table 1.** Examples of variation in sex determination mechanisms within species c.q. between sister species.

Species name (class, order)	Sex: determination variants	Distribution	Effect of temperature on sex determination	Genetic basis known	References
*Bufo* spp. (Amphibia; Anura)	XY, ZW	*B. bufo* ZW, *B. spinosus* XY	Plausible^a^	No	(Dufresnes et al., 2020)
*Bufo viridis* (Amphibia; Anura)	XY, ZW	Asia Minor XY, Moldavia ZW	Plausible^a^	No	(Odierna et al., 2007; Stöck et al., 2013)
*Cyrtodactylus* spp. (Reptilia; Squamata)	XY, ZW	*C. chaunghanakwaensis* XY, *C. pharbaungensis* ZW	Plausible^a^	No	(Keating et al., 2021)
*Eleutherodactylus* spp. (Amphibia; Anura)	XY, ZW	*E. euphronides* and *E. shrevei* ZW, *E. maussi* X_1_X_2_Y	Plausible^a^	No	(Schmid et al., 2002a; b)
*Gambusia* spp. (Actinopterygii; Cyprinodontiformes)	XY, ZW	*G. affinis* ZW, *G. holbrooki* XY	Plausible^a^	No	(Kottler et al., 2020)
*Gekko* spp. (Reptilia: Squamata)	XY, ZW, TSD	*G. gecko* XY, *G. japonicus* XY/TSD, *G. hokouensis* ZW	TSD present in *G. japonicas*	No	(Gamble, 2010)
*Glandirana rugosa* (Amphibia, Anura)	XY (2), ZW (2)	North/central Japan: ZW; east/west XY	Plausible^a^	No	(Ogata et al., 2018)
*Hemidactylus* spp. (Reptilia: Squamata)	XY, ZW	*H. turcicus* and *H. mabouia* XY, *H. frenatus* ZW	TSD inferred as ancestral state	No	(Gamble et al., 2015)
*Hypoatherina tsurugae* (Actinopterygii; Atheriniformes)	XY, TSD	-	Masculinizing in XX individuals	Partial (candidate master gene: *amhy*)	(Miyoshi et al., 2020)
*Musca domestica* (Insecta, Diptera)	XY (multiple), ZW	Low latitude ZW, high latitude XY	Feminizing^b^	Yes	(Hamm et al., 2015)
*Oryzias* spp.	XY, ZW	*O. latipes* and *O. dancena* XY, *O. hubbsi* and *O. javanicus* ZW	Masculinizing (in XX individuals in *O. latipes*)	Partial (*DMY* master sex determining gene in *O. latipes*, but not others)	(Sato et al., 2005; Takehana et al., 2007, 2008)
*Pogona vitticeps* (Reptilia; Squamata)	ZW, TSD	-	Feminizing	Partial (candidate master gene: *nr5a1*)	(Holleley et al., 2015; Zhang et al., 2022)
*Rana temporaria* (Amphibia; Anura)	XY (multiple)	Low latitude X-Y diverged, high latitude X-Y homomorphic	Masculinizing	Partial (candidate master gene: *Dmrt1*)	(Rodrigues et al., 2014)
*Scincella* spp. (Reptilia: Squamata)	XY, ZW	*S. assata* and *S. cherriei* XY, *S. lateralis* X_1_X_2_Y *S. melanosticta* 2n=30 ZW	Plausible^a^	No	(Castiglia et al., 2013; Mezzasalma et al., 2021; Patawang et al., 2018)

*Note*. Divergent evolution of sex determination mechanisms within a single species may occur as different populations adapt to different environmental conditions as explained by our model. Similarly, closely related species may exhibit different sex determination mechanisms when occupying different habitats. These species c.q. species complexes may provide opportunities to validate our model or aspects thereof, though a lack of knowledge on the genetic basis of sex determination and/or effect of environmental variation may need to be resolved to do so.

^a^Temperature effects on sex determination are described in related species (same order or genus),

^b^Temperature effect theorized, but lacking empirical validation.

## Methods

Here, we briefly describe the model; a more detailed description of the model and simulation techniques is provided in the Supplementary Methods. We developed an individual-based simulation model, where individuals occupy a linear array of subpopulations (demes) arranged along a temperature gradient. The life cycle is as follows: adults reproduce sexually and then die; their offspring undergo sexual development and viability selection; a fraction of the surviving offspring migrates from their natal subpopulation to a neighboring subpopulation; finally, individuals mature and the next round of reproduction begins.

Motivated by the SD mechanisms of the housefly (Box 1), individual sexual development was modeled to result from interactions between a single feminizing gene *F*, one or more masculinizing genes *M* and the local temperature of an individual’s environment; note that labels such as “female” and “feminizing” are interchangeable for “male” and “masculinizing” to suit other SD mechanisms, e.g., ZW systems instead of XY systems. The *F* gene produces a temperature-dependent amount of product which is partially inhibited or degraded by the products from the *M* genes; the remaining or net amount of *F* product, denoted by ${\hat{z}}_{F}$, determines sex: if ${\hat{z}}_{F}$ exceeds a threshold value $\theta_{F}$, the individual develops as female, whereas if ${\hat{z}}_{F}$ is below a second threshold value $\theta_{M}<\theta_{F}$ it develops into a male. If ${\hat{z}}_{F}$ is between the two thresholds, $\theta_{M}\leq {\hat{z}}_{F}\leq \theta_{F}$, then the individual develops into an infertile intersex.

The value of ${\hat{z}}_{F}$ is obtained by summing up the net expression levels $z_{F}$ of both *F* alleles in an individual. The quantitative value of $z_{F}$ can vary between *F* alleles and depends on (1) the temperature-dependent expression level of the allele, (2) the allele’s sensitivity to *M* product, and (3) the amount of *M* product. Specifically, normalized temperature varies between T=0 at one end (the “north”) of the array of subpopulations and T=1 at the other end (the “south”) and it affects the net amount of *F* product according to


$$\begin{array}{l} z_{F}=(z_{F_{0}}\left(1+\beta T\right)+\varepsilon )(1-s_{F_{M\;}}{\hat{z}}_{M}) \end{array}$$ (1)

The first factor on the right, $z_{F_{0}}\left(1+\beta T\right)+\varepsilon$, represents the initial amount of *F* product, before partial degradation by *M* product, where $z_{F_{0}}$ is the *F* allele’s baseline expression level at T=0, β≥0 quantifies the linear rate of increase in *F* expression with temperature, and ε represents random variation in expression levels due to environmental and/or developmental noise. The second factor, $1-s_{F_{M}}{\hat{z}}_{M}$, represents the proportion of *F* product that is not degraded by *M* product, where $0\leq s_{F_{M}}\leq 1$ is the *F* allele’s sensitivity to *M* product and ${\hat{z}}_{M}$ is the cumulative amount of *M* product produced by the individual’s *M* alleles.

The baseline expression level $z_{F_{0}}$ and sensitivity $s_{F_{M}}$ of *F* alleles are evolvable trait values, as are the expression levels of *M* alleles. Whenever a gamete is produced, each allelic trait incurs a mutation with a certain probability and its trait value is modified. Most mutations are “regular” mutations that modify traits by adding a small Gaussian amount with mean zero, but a small fraction of mutations are “null mutations” such that the resulting trait values (allelic expression levels or sensitivity to *M* product) are zero and cannot evolve any further. See Supplementary Table 2 for all model parameters and their default values used in the simulations.

The initial populations all have an XY male heterogametic system: all individuals carry two *F* alleles on an autosomal *F* locus and males additionally carry a single *M* allele (designated *M*_Y_) on their Y-chromosome. Initially there are no *M* alleles on autosomes, but we assume that during meiosis sometimes a new *M* allele (designated *M*_A_) is created on an autosomal locus; this is assumed to occur via de novo evolution of a novel *M* allele, but can also occur via transposition from a Y-chromosomal *M* allele. Individuals carry at most four *M* alleles: two on the Y-chromosome (if they have two Y chromosomes) and two on an autosome. In natural systems, many loci may be capable of evolving a male-determining function (Bopp, 2010), but we consider here only a single autosomal locus for simplicity. Thus, the initial XY population has the potential to evolve into a population with a new system of male heterogamety where an autosomal chromosome with an *M* allele has replaced the original Y-chromosome. Evolution of populations with female heterogamety is also a potential outcome, if females become heterozygous for an insensitive *F* allele (i.e., with $s_{F_{M}}=0$) with a sufficiently high expression level.

We also allowed for Y-chromosomal fitness effects: (1) individuals homozygous for the Y-chromosome will have reduced viability $0\leq s_{YY}\leq 1,$ and (2) Y-chromosomes carry sexually antagonistic alleles that are beneficial to males and detrimental in females, with additive effect $1+s_{a}$ males and $\;1-s_{a}$ in females (where $s_{a}\geq 0$ is the antagonistic effect). The combined effects of Y-homozygosity and sexual antagonism are assumed to be multiplicative, i.e., a male with two Y-chromosomes will have his expected fitness modified by a factor $s_{YY}(1+s_{a})$, whereas a female with two Y-chromosomes gets $s_{YY}(1-s_{a})$.

## Results

### *F* activity relative to SD thresholds determines which SD systems are evolutionary stable

As an initial exploration of our model, we performed a set of 10,000 simulations without temperature-dependent overexpression, but for different values for the SD thresholds $\theta_{M}$ and $\theta_{F}$, as well as different *M*_A_ activation rates $\mu_{D}$ (sampled from a uniform distribution with range [0, 0.05]). Here, de novo activation of *M*_A_ causes a male-biased sex ratio, thereby promoting the invasion of female-determining alleles (Bull & Charnov, 1977; Wilkins, 1995), similar to, e.g., sex chromosome meiotic drive (Jaenike, 2001; Kozielska et al., 2010). In addition, for these simulations, we did not incorporate fitness effects associated with *M*_Y_. For each simulation, we determined the most prevalent genotype among females and males at equilibrium, based on the expression and sensitivity levels of their *F* alleles as well as the number of expressed *M*_Y_ and *M*_A_ alleles. We categorized simulations according to the activity of a single *F* allele ($z_{F}$) relative to $\theta_{M}$ and $\theta_{F}$ to determine which SD systems can evolve under different levels of *F* activity.

We find that under different relationships between the maximum potential activity of a single *F* allele ($z_{F}$) and the SD thresholds, different SD systems can evolve (Supplementary Table 3). When $\theta_{M}<z_{F}<\theta_{F}$, nearly all simulations yield an SD system where both sexes have two sensitive and expressed *F* alleles, and males are heterozygous for a single *M* (either *M*_Y_ or *M*_A_; females *F/F; +/+*, males *F/F; +/M*, with + indicating the absence of an expressed *M* allele). In contrast, when $z_{F}<\theta_{M}<\theta_{F}$ or $\theta_{M}<\theta_{F}<z_{F}$, we additionally encounter systems in which *F* alleles evolved to become insensitive and/or unexpressed, but the distribution of these alleles over the sexes differs between these two scenarios: when $z_{F}<\theta_{M}<\theta_{F}$, females carry two insensitive *F* alleles, whereas males carry a single insensitive *F* and one unexpressed *F*; here, insensitive *F* alleles can be regarded as recessive female-determining alleles, whereas the sensitive and expressed *F* allele (in presence of *M*) or unexpressed *F* plays the role of a dominant male-determining allele. When $\theta_{M}<\theta_{F}<z_{F}$, females may carry a single insensitive *F* allele, whereas males carry none, suggesting that insensitive *F* alleles are dominant female-determining alleles. This is corroborated by the presence of expressed *M* alleles in both sexes in the simulations, which would otherwise induce maleness in their carriers.

**Figure 2.**
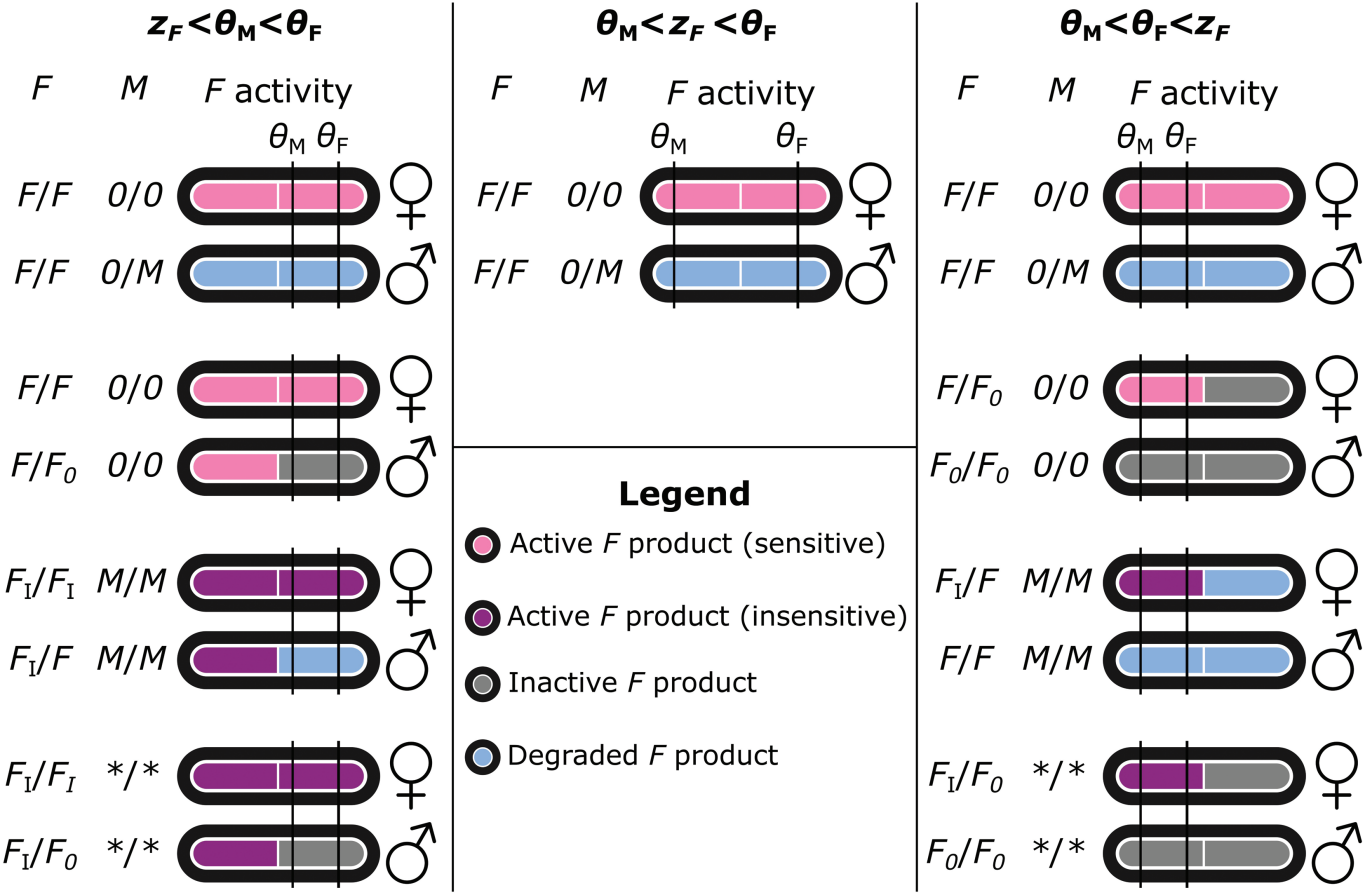
Classification of sex determination systems depending on relative values of thresholds and *F* activity. Each system is defined by a recurrent pair of female and male genotypes that can only generate those two genotypes (conform Bull & Charnov, 1977). Here we define three alleles for locus *F*: a regular *F* that is expressed and sensitive to *M*; a variant *F*_I_ that is insensitive to *M* and expressed; and a variant *F*_*0*_ that is unexpressed. For *M*, we distinguish between active (*M*) and inactive (*0*) variants. We use $z_{F}$ to refer to the activity of a single *F* allele. In systems with both *F*_I_ and *F*_*0*_, *M* has no function and may be present or absent; this is indicated by asterisks (*/*).

In simulations where insensitive *F* alleles have evolved, we find that the remaining *F* alleles have become unexpressed, and in some cases that expressed *M* alleles are lost as well. Here, insensitive *F* alleles spread first, along with an increased frequency of expressed *M* alleles in both sexes (Supplementary Figure 1), so that an equal sex ratio is maintained. Next, sensitive *F* alleles become unexpressed (and subsequently insensitive due to the constant mutation pressure affecting *F* sensitivity); the insensitive expressed *F* allele is retained as it now performs the SD function. Along with the increase in unexpressed *F* alleles, M alleles similarly become unexpressed. Based on these dynamics, we infer that evolution of *F* insensitivity indirectly renders the loss of expression selectively neutral for sensitive *F* alleles, which in turn renders the loss of *M* expression neutral; both genes may therefore decay via mutation accumulation in a stepwise order (Supplementary Figure 2). In addition to the systems identified in our simulations, we speculate some other systems may also be evolutionary stable (Figure 2). Their absence from our simulations may be because they only rarely arise through evolution, or because they represent intermediate states between some of the systems that are observed. The latter for example applies to systems where females have one insensitive and one sensitive F allele, males have two sensitive F alleles, and *M* is fixed in both sexes (females *F*_I_*/F; M/M*, males *F/F; M/M* in Figure 2). This system is prevalent in some simulations prior to the accumulation of unexpressed *F* alleles and loss of *M* (e.g., Supplementary Figure 1). In absence of fitness effects related to *M*_Y_, recurrent de novo mutation of *M*_A_ results in *M*_A_ replacing *M*_Y_ as the male-determining factor; without evolution of *F*, this represents a transition from one male heterogamety system to another, as *M*_Y_ and *M*_A_ perform equivalent functions. Male heterogamety systems with *M*_Y_ or *M*_A_ as the male-determining gene are observed regardless of the activity of *F* relative to the SD thresholds (Supplementary Table 3). Moreover, male heterogamety with *M*_Y_ or *M*_A_ as a dominant male-determiner is observed in all simulations when $\theta_{M}<z_{F}<\theta_{F}$, suggesting this is the only stable SD system under these conditions, so that insensitive *F* alleles cannot be part of any SD mechanism under these conditions.

### Evolution of *F* insensitivity and establishment of SD system gradients

The above results show how *F*, *M*, and the SD system as a whole evolve in relation to the SD thresholds $\theta_{F}$ and $\theta_{M}$. Most importantly, we find that *F* can evolve into a dominant female-determining gene by becoming insensitive to *M*, provided that a single *F* allele generates enough *F* product to induce feminization, i.e., the threshold for feminization $\theta_{F}$ is sufficiently low relative to *F* expression $z_{F}$. We refer to such insensitive dominant feminizing *F* alleles as *F*_I_. Invasion of *F*_I_ alleles in our model is primarily driven by the *de novo* evolution of *M*_A_, which causes a slight male-biased sex ratio, thereby causing feminizing mutations to be favored (but see Discussion) (Bull & Charnov, 1977). Provided that the feminization threshold $\theta_{F}$ is constant under all conditions, local variation in temperature could then lead to divergent evolution of SD systems as temperature effects on *F* expression could then allow for local evolution of a female heterogamety system.

**Figure 3.**
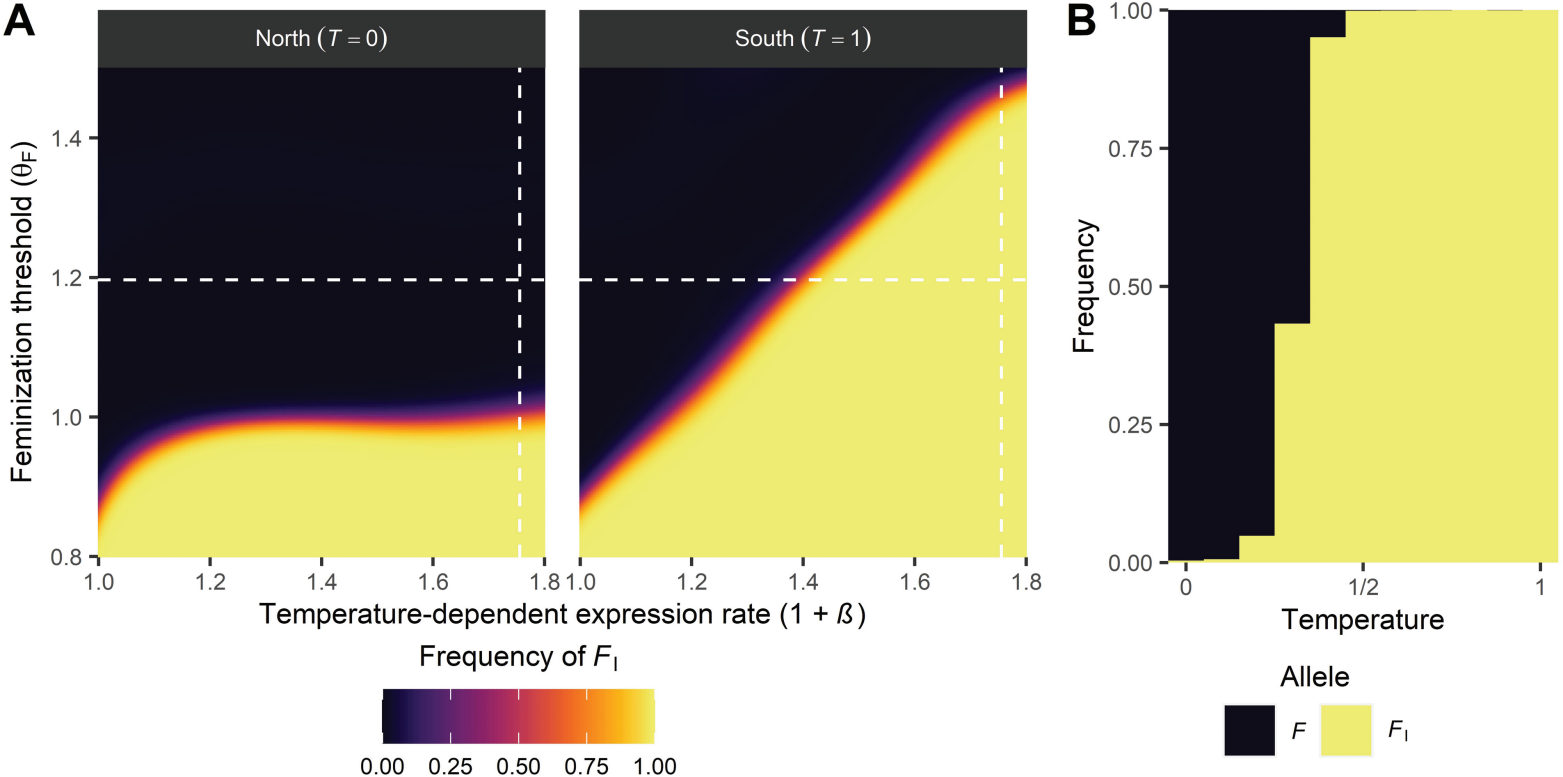
Conditions for spread of a dominant female-determining gene *F*_I_ as a function of the temperature-dependent expression rate 1+β and the feminization threshold $\theta_{F}$. When β=0, expression is unaffected by temperature. (A) Equilibrium frequencies of *F*_I_ at edges (first/last deme) of the population range; local temperatures are indicated in brackets (parameter values: $\theta_{M}=0.2;\mu_{D}=0.001$). In the northern deme (T=0), temperature-dependent expression of *F* is lowest while expression is highest in the southern deme (T=1). (B) Between these two extremes, the equilibrium frequency of *F*_I_ increases along the temperature gradient (shown here for $\theta_{F}=1.20;\theta_{M}=0.2;\beta =0.76;\;\mu_{D}=0.014$; the results depicted were chosen based on whether or not a gradient in *F*_I_ was observed, with parameter values in each simulation being chosen at random from uniform distributions). Depicted in A and B are the frequency of the *F*_I_ allele at the maternally-inherited locus in females. White dashed lines in A indicate the parameter values for the simulation results depicted in B (vertical line: 1+β; horizontal line: $\theta_{F}$).

To test this reasoning, we used simulations in which we varied two parameters: the rate β at which temperature increases *F* expression (see Equation 1) and the threshold value $\theta_{F}$ for feminization. Here, we exclude Y-chromosomal fitness effects, but explore their impact on SD evolution in the following section. We found that *F*_I_ can spread in the entire metapopulation when the feminization threshold $\theta_{F}$ is sufficiently small (Figure 3A), regardless of how strongly temperature affects *F* expression. For sufficiently high values of $\theta_{F}$, *F*_I_ was unable to spread in colder demes because it would result in intersexual development (Supplementary Figure 3), but could still spread in warmer demes. Under these conditions, a geographical cline in the frequency of *F*_I_ evolves (Figure 3B).

These results underline that the distribution of *F*_I_ is constrained by the expression level of *F*, and show that temperature-dependent effects on gene expression can establish gradients when temperature varies. Due to its feminizing effect, an *F*_I_ allele is transmitted as if it were a female-limited W-chromosome in a ZW system, wherein males have a ZZ genotype, whereas females have a ZW genotype (in contrast to XY systems where females are XX and males are XY). As a result, it occurs only in *F*_I_/*F* females (or non-reproducing intersexes). In the presence of *M* product, the *F*_I_ product is not broken down but the product of regular sensitive *F* alleles is degraded, so that regular *F* alleles do not contribute to the total *F* product. This scenario becomes increasingly probable as either *M*_Y_ and/or *M*_A_ frequency increases, as more *F*_I_-bearing individuals will also bear *M*_Y_ and/or *M*_A_. Therefore, feminization of developing embryos under these conditions is achieved solely by the activity of the *F*_I_ allele. Because *F*_I_ is insensitive to *M*, the total *F* activity is determined by its expression (see Equation 1). When no temperature-dependent expression occurs, feminization is only achieved when the baseline genetic expression level exceeds the feminization threshold, but in the presence of temperature effects is less constrained. Therefore, when $\theta_{F}$ is sufficiently low, *F*_I_ can spread everywhere independent of temperature (Figure 3A, left panel), whereas otherwise *F*_I_ frequencies depend on the temperature-dependent expression rate (Figure 3A, right panel) and the local temperature (Figure 3B).

### Y-chromosomal fitness effects modulate the conditions under which SD turnover can occur and can enable complex SD polymorphisms

For the simulations discussed above, we assumed that *M*_Y_ is not associated with any fitness effects. Under these conditions, *M*_Y_ was always lost due to de novo evolution of *M*_A_, which causes a male-biased sex ratio so that both *M*_Y_ and *M*_A_ are selected against. Recurrent mutation of *M*A ensures that it nonetheless remains present in the population, but this does not hold for *M*_Y_; in absence of any associated selective effect, *M*_Y_ is therefore purged. However, as *M*_Y_ is the ancestral SD gene, it may have induced the chromosome on which it is located to undergo Y-chromosome evolution (reviewed in Bachtrog, 2013; Schenkel & Beukeboom, 2016). If so, this chromosome is expected to become enriched for sexually antagonistic (SA) genetic variants as well as recessive deleterious mutations. In effect, this would cause *M*_Y_ to be associated with higher fitness in heterozygous *+/M*_Y_ males, but to induce a fitness cost to *M*_Y_-bearing females (who also carry *F*_I_) as well as all homozygous *M*_Y_/*M*_Y_ individuals. Both sexually antagonistic genetic variation and a cost of homozygosity can in theory strongly affect evolutionary transitions in SD (reviewed in van Doorn, 2014). We therefore performed additional simulations where we considered a sexually antagonistic fitness effect of the Y-chromosome (see Supplementary Table 1), causing the Y-chromosome to affect individual fitness during the mating phase or during development (positively in males, negatively in females). In addition, we include costs of *M*_Y_/*M*_Y_ homozygosity as a form of viability selection during embryogenesis.

**Figure 4.**
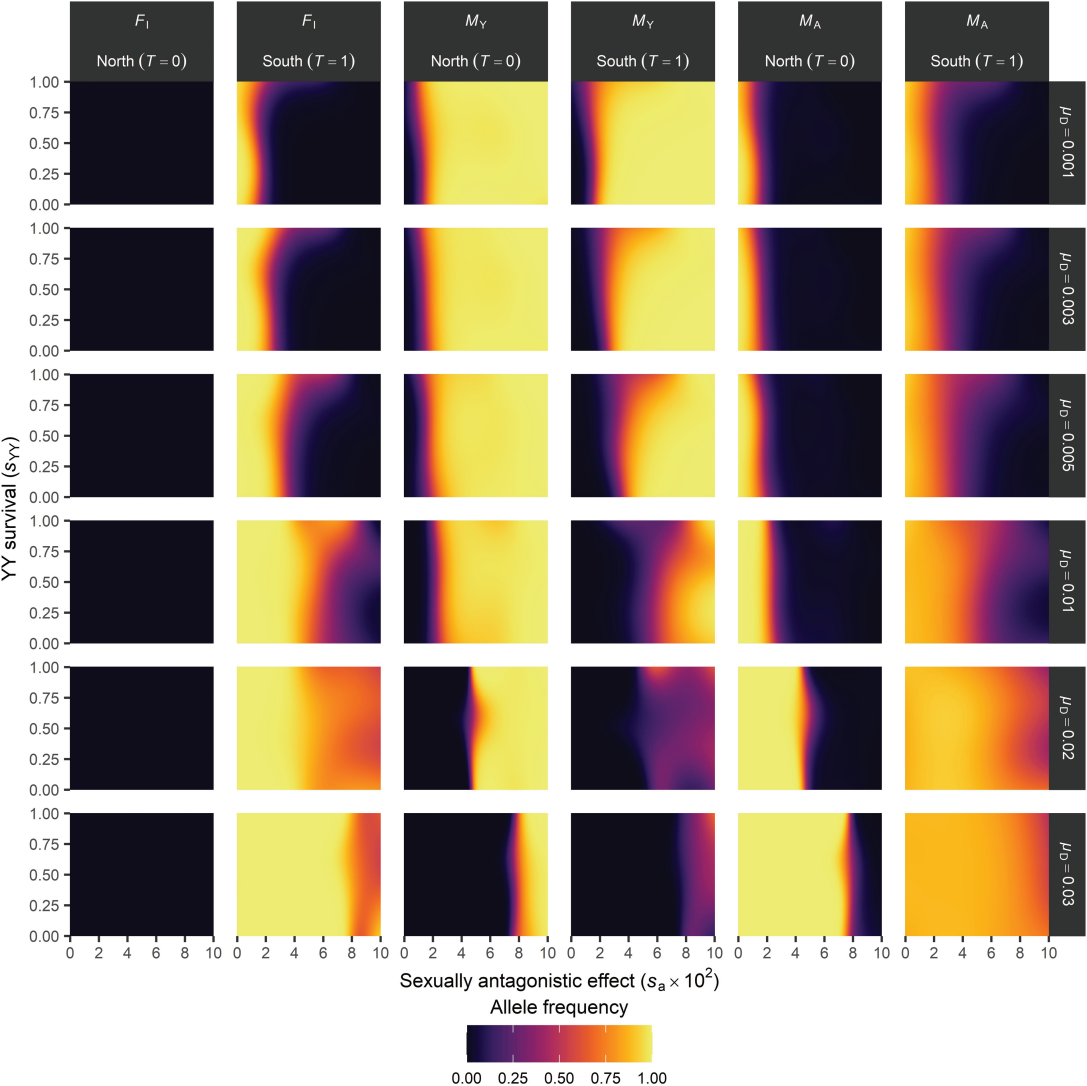
Y-chromosomal fitness effects alter the scope for SD transitions. Shown are the predicted equilibrium frequencies of *F*_I_ in females (maternally inherited alleles), *M*_Y_ and *M*_A_ in males (paternally inherited alleles); we restrict our analysis to the maternal (*F*_I_) c.q. paternal (*M*_Y_, *M*_A_) alleles to account for their (potential) sex-specific transmission. Horizontal labels indicate locus and temperature, vertical labels the *M*_A_ activation rate $\mu_{D}$. Further parameter values used in simulations: $\theta_{F}=1.2;\theta_{M}=0.3;\beta =0.5$. Predicted allele frequencies were smoothed with binomial generalized additive models.

When *M*_Y_ is under sexually antagonistic selection, we find that it is maintained over *M*_A_ (Figure 4). Sexually antagonistic selection on *M*_Y_ can also inhibit invasion of *F*_I_ if selection is sufficiently strong, so that negative effects of *M*_Y_ in females reduces the fitness of *F*_I_/*F* females relative to non-*M*_Y_-carrying *F/F* females. However, when the rate of introduction of novel *M*_A_ alleles is sufficiently high, *F*_I_ can always invade even if the selective effects associated with *M*_Y_ are strong. When *M*_A_ originates more frequently and therefore reaches higher frequencies, a more male-biased sex ratio results (similar to Y-chromosomal meiotic drive; Jaenike, 2001; Kozielska et al., 2010) and hence the selective benefit for *F*_I_ as a female-determining factor increases.

Y-chromosomal fitness effects can also enable maintenance of both *M*_Y_ and *M*_A_ in the population, albeit in different locations. This occurs when *M*_Y_ is disfavored through reduced survival of YY homozygotes in subpopulations with *F*_I_, in contrast to subpopulations without *F*_I_, where *M*_Y_ is favored over *M*_A_ via sexually antagonistic selection in +/*M*_Y_ heterozygotes. Such *M*_Y_ versus *M*_A_ polymorphism is highly similar to the distribution of Y-chromosomal versus autosomal *M*-factors in the housefly *M. domestica*. In this species, autosomal *M*-factors are more prevalent at lower latitudes and coincide with a dominant feminizing allele *tra*^D^ (which is equivalent to *F*_I_ as described above), resulting in three latitudinal gradients in Y-chromosomal *M*-factors, autosomal *M*-factors, and the *tra*^D^ allele. We find that Y-chromosomal fitness effects enable the evolution of this complex system in our model (see Box 1). This provides an adaptive explanation for the evolution of this system in contrast to existing models of SD evolution, which have been unable to predict when stable polymorphisms for *tra* versus *tra*^D^ along with Y-chromosomal *M*-factors versus autosomal *M*-factors may occur in general, and in particular when these coincide along environmental clines as observed under natural conditions (e.g., Kozielska et al., 2006, 2010, but see Meisel et al., 2016, for an alternative explanation that invokes linkage between different SD genes and sexually antagonistic loci).

**Box 1. Evolution of the polymorphic housefly system**

Our model has been inspired by the common housefly *Musca domestica*, in which wild populations harbor different SD systems (reviewed in Hamm et al., 2015). Here, we discuss how our model can explain the evolution of this system.

In houseflies, sex is determined by a linear cascade of genes. First, *transformer* (*tra*) induces female development when active in developing embryos (Hediger et al., 2010). Its activity depends on an autocatalytic feedback loop where female-specific TRA^F^ protein directs the splicing of Tra pre-mRNA into the female-specific Tra^F^ mRNA, thereby ensuring the production of novel TRA^F^ protein to maintain the loop. Possibly, temperature effects on *tra* work by affecting pre-mRNA splicing, as splicing is sensitive to temperature as well as other stressors (Palusa et al., 2007). Second, masculinizing factors (*M*-factors) such as *Mdmd* (Sharma et al., 2017) trigger male development by inhibition of the *tra* loop through splicing of *tra* pre-mRNA to a male-specific variant. Intriguingly, the *M*-factors in *M. domestica* are found on different chromosomes in different populations; most of these correspond to orthologs of *Mdmd*, and the genomic regions harboring these *M*-factors consist of multiple (partial) repeats of the *Mdmd* gene, suggesting that these have originated through gene duplication and/or translocation (Sharma et al., 2017, Li et al., in prep). There also exists an insensitive variant of *tra*, *tra*^D^, that induces female development in all carriers regardless of whether they carry any *M*-factors. In our model, *F* represents *tra* and likewise *tra*^D^ corresponds to the dominant *F*_I_ as discussed in the main text; *M*_Y_ and *M*_A_ in our model represent *M*-factors.

High-latitude *M. domestica* populations have a male heterogamety (XY) system in which the Y-chromosomal *Mdmd* gene induces maleness, and all individuals carry two regularly sensitive *tra* alleles. At lower latitudes, however, females usually carry the insensitive *tra*^D^ allele, and both sexes can be homozygous for an autosomal copy of *Mdmd*; hence, these populations have a female heterogamety (ZW) system (Supplementary Figure 4; Hamm et al., 2015; Kozielska et al., 2008). The geographical transition between these two SD systems is gradual, so that clines exist in the frequencies of Y-chromosomal *Mdmd* (decreasing toward lower latitudes), autosomal *Mdmd* and *tra*^D^ (both increasing toward lower latitudes). Given that a single *tra*^*D*^ allele is sufficient for feminization (even in the presence of multiple M-factors; Hamm et al., 2015) suggests that at least in natural populations it would mimic an *F*_I_ allele with a net expression level $z_{F}$ that exceeds $\theta_{F}$. Therefore, in our model this system would likely mimic a transition from $\theta_{M}<z_{F}<\theta_{F}$ to $\theta_{M}<\theta_{F}<z_{F}$. Temperature likely plays a causal role in maintaining these gradients by affecting the SD process (Adhikari et al., 2021; Delclos et al., 2021; Feldmeyer, 2009; Feldmeyer et al., 2008). Temperature effects on housefly SD have been reported in the form of biased sex ratios produced in wildtype crosses (Feldmeyer, 2009) as well as in females carrying the *masculinizer* (*man*) mutation, another variant of *tra* (Hediger et al., 2010; Schmidt et al., 1997). The *man* mutation represents a maternal-effect male-determining gene, where *man*-carrying females can produce all-male progeny even if the progeny do not carry an *M*-factor. However, this effect is incomplete and temperature-sensitive (Schmidt et al., 1997), with offspring sex ratios more male-biased at higher temperatures. Altogether, temperature seems to have an important influence on SD in *M. domestica*, but the underlying mechanisms are not yet fully understood.

**Figure 5.**
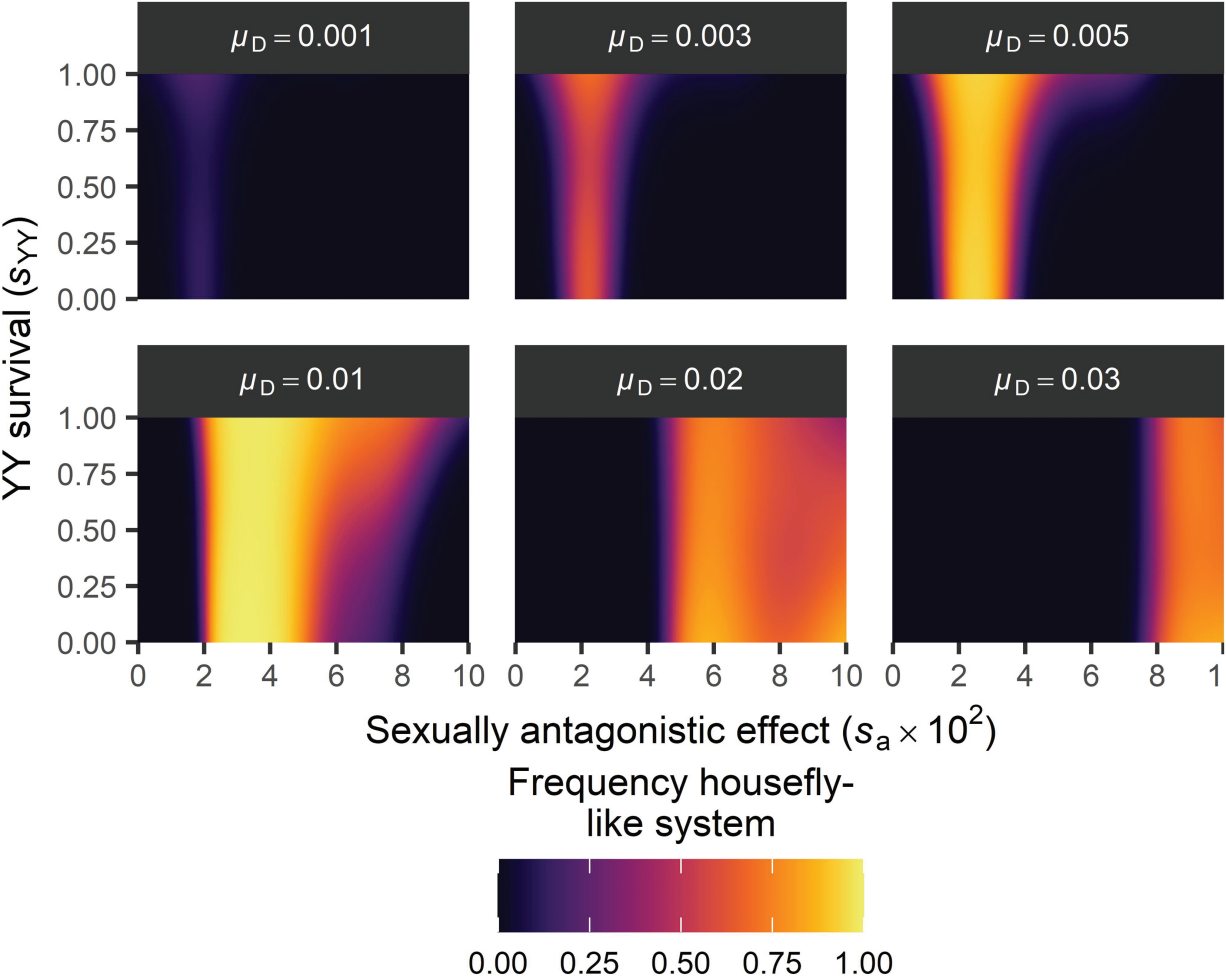
Evolution of a housefly-like SD system. A housefly-like SD system is defined by *M*_Y_ being the major allele at T=0 but the minor allele at T=1 (in males, paternally inherited allele), and vice versa for *M*_A_ (in males, paternally inherited alleles) and *F*_I_ (in females, maternally inherited alleles). Frequency denotes the predicted frequency of observing a housefly-like system at equilibrium in the model. Parameter values: $\theta_{F}=1.2;\theta_{M}=0.3;\beta =0.5$. Simulations were scored as exhibiting a housefly-like system as described above (10,000 simulations in total). To obtain these predicted scores, we fitted a binomial generalized additive model.

**Figure 6.**
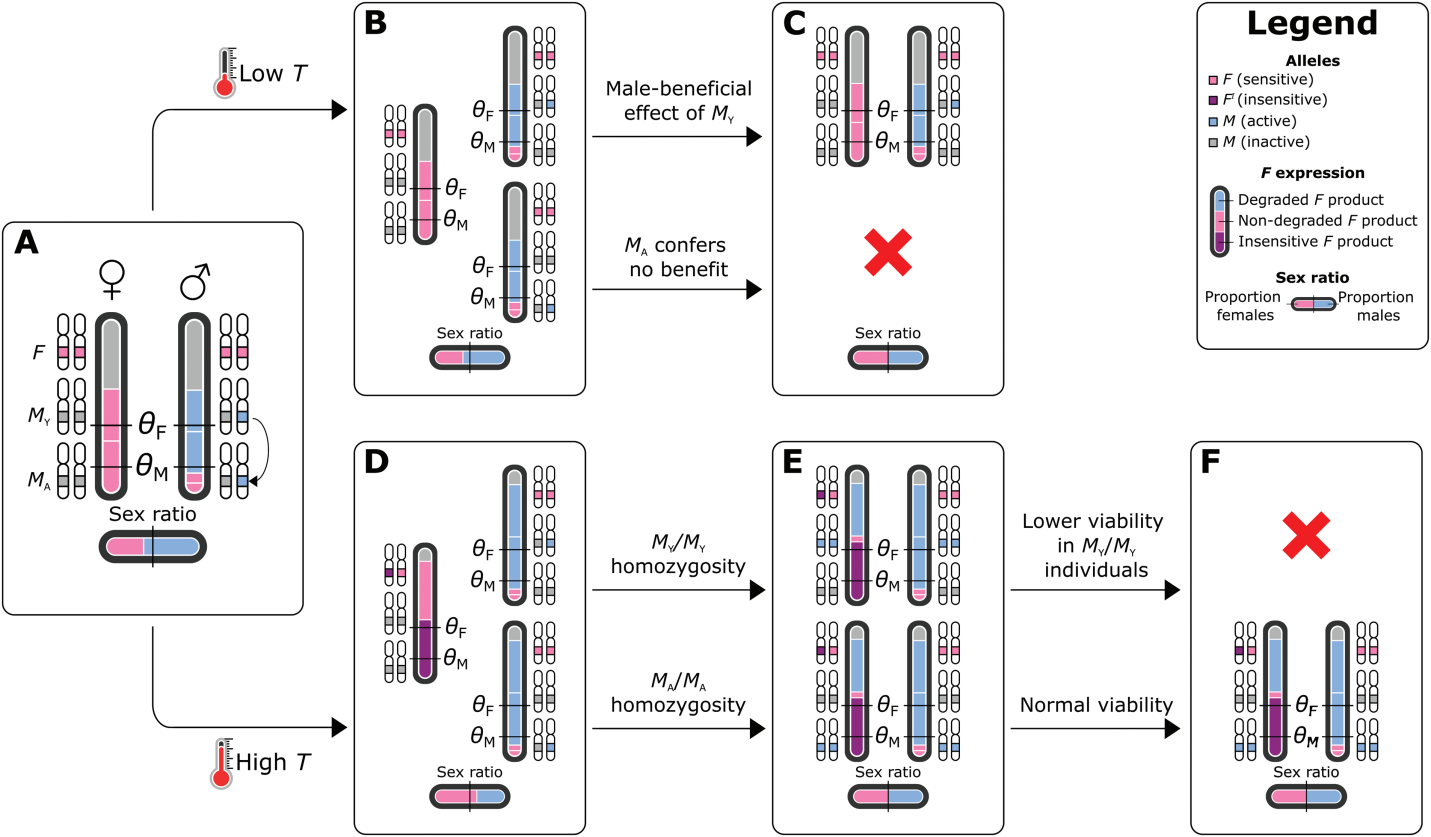
A novel hypothesis for the evolution of the housefly polymorphic sex determination mechanism. (A) Evolution of *M*_A_ (here represented by transposition of *M*_Y_) results in an excess of *M* factors in the population and a male-biased sex ratio. (B) At low temperatures, the *M*-insensitive *F*_I_ allele (equivalent to *tra*^D^ in *M. domestica*) cannot evolve, and both *M*_Y_ and *M*_A_ persist in a heterozygous state. (C) Because *M*_Y_ is associated with a fitness benefit in males, *M*_Y_-bearing males outcompete *M*_A_-bearing males, resulting in a return to the ancestral state with *M*_Y_ as the sole male-determining allele in a XY system. (D) In contrast, at high temperatures, the *F*_I_ allele can evolve, and has a fitness benefit as a result of sex ratio selection. (E) As *F*_I_ spreads, *M* alleles can be transmitted by females resulting in the formation of homozygous *M*_Y_/*M*_Y_ and *M*_A_/*M*_A_ individuals. (F) Because *M*_Y_ homozygosity is associated with a viability cost, these individuals have lower fitness than *M*_A_/*M*_**A**_ individuals. This results in a loss of the *M*_Y_ allele and fixation of the *M*_A_ allele in its place as a co-factor for male development. In effect, a transition has occurred from XY male heterogamety to ZW female heterogamety as the sex-determining role has been taken over by *F*_I_.

The housefly with its different SD systems is represented in our model by gradients in the frequencies of *M*_Y_ (decreasing with temperature), *M*_A_, and *F*_I_ (both increasing with temperature). Presumably, *tra*^D^ is limited to warmer localities for similar reasons as *F*_I_ in our model, i.e., because a single *tra*^D^ allele may not be sufficient to induce feminization at low temperatures. Y-chromosomal *Mdmd* and autosomal *Mdmd* may follow similar dynamics as *M*_Y_ and *M*_A_. Genomic analyses of the *Mdmd* loci on the Y-chromosome and various other chromosomes suggests that *Mdmd* may exhibit (or have exhibited) transposon-like activity, leading to tandem duplications of transposition of these duplications as a complex to other chromosomes (Sharma et al., 2017, Li et al., in prep). Y-chromosomal fitness effects can yield gradients in *M*_Y_ and *M*_A_, but may also prevent the spread of *F*_I_, particularly when novel *M*_A_ alleles enter the population at a low rate. The evolution of a housefly-like polymorphic SD system therefore depends on a balance between the Y-chromosomal fitness effects and the rate at which new autosomal *Mdmd* arises. In our model, we find that a housefly-like system can evolve under various rates of *M*_A_ de novo evolution (Figure 5). Higher rates of *M*_A_ evolution require stronger SA effects for *M*_Y_ to be maintained in low-temperature demes. Costs of YY homozygosity do not appear essential for *M*_Y_ to be lost in the presence of *F*_I_, although they may increase the likelihood of *M*_Y_ being lost in favor of *M*_A_ in the presence of *F*_I_ by reducing the fitness of YY homozygotes. Possibly, costs of *M*_Y_ in females due to sexually antagonistic selection may suffice to promote the loss of *M*_Y_. Our model therefore can explain the evolution of complex SD systems such as those found in houseflies. Thus, we propose a novel hypothesis for the evolution of the housefly system in which the sex determination cascade is shaped by a combination of environmental influences on *tra*, recurrent evolution of autosomal *Mdmd*, and fitness effects associated with the Y-chromosomal copy of *Mdmd* (Figure 6).

This hypothesis, along with the results in this manuscript, highlights several promising aspects of housefly SD and its evolution that require further investigation. First and foremost, the effect of temperature on *tra* functionality needs to be verified, as current data are inconclusive (Adhikari et al., 2021; Feldmeyer, 2009). Our model predicts that *tra* function is enhanced at high temperatures, allowing for the evolution of a dominant feminizing allele *tra*^*D*^. Given that *tra* regulates its own activity via an autoregulatory feedback loop (Dübendorfer et al., 2002; Hediger et al., 2010), this enhanced function may be effectuated in a variety of manners, including pre-mRNA expression, mRNA splicing, and protein catalytic activity, so that a purely molecular analysis of *tra* function may be inconclusive. These issues are aggravated by the fact that whether or not the loop is established is typically determined in the zygotic stage, so that analyses of *tra* function in the adult stage may not be informative (in contrast to Adhikari et al., 2021). Given that *tra* is expressed even in presence of *Mdmd*, it seems likely that the hypothesized gain-of-function of *tra*^*D*^ must act post-transcriptionally. Instead, an inverse approach may be more fruitful; if *tra*^*D*^ can only evolve at high temperatures, then *tra*^*D*^-bearing flies reared at low temperatures should be more prone to sex reversal or intersexuality. Second, the evolution of *Mdmd* must be further explored to evaluate the transpositional activity of *Mdmd* itself or the supergene complex containing this gene. The complex arrangement of *Mdmd* copies in *M*-loci strongly suggests *Mdmd* may at one point have transposed frequently (Li et al., in prep), but it is undetermined whether this is still the case. Third and final, fitness assays of houseflies with Y-chromosomal versus autosomal *Mdmd* must be made to determine if there are any fitness effects linked to these genes. In vitro studies on houseflies strains that carry *Mdmd* on different chromosomes found that the *Mdmd*-bearing chromosome may exhibit some genetic differentiation (Meisel et al., 2020), and that these may be associated with fitness effects (Son et al., 2019). In vivo characterization is however required to validate these effects, and to determine their impact on the maintenance of *M*_Y_ over *M*_A_ as predicted in our model.

## Discussion

We have presented a model for the evolution of SD systems in a context where sex is determined by genetic factors in combination with environmental effects. In our model, female development is induced when the activity of a feminizing gene *F* exceeds a certain threshold, whereas male development is achieved when *F* activity is below a different and lower threshold. This can be caused by inhibition of *F* activity by the maleness-promoting gene(s) *M*_Y_ and/or *M*_A_, or by loss of *F* expression. We incorporated a positive effect of temperature on the expression of a feminizing locus *F*. We find that several different SD systems can be realized depending on the activity of an *F* allele relative to the threshold values for masculinization and feminization. Temperature-dependent effects on *F* expression may alter the relationships between *F* activity and SD thresholds, thereby enabling the evolution of different genetic SD systems. A particular prediction is the transition from male heterogamety to female heterogamety, which occurs in our model via the evolution of an insensitive dominant feminizing variant (*F*_I_) that induces femaleness even in the presence of *M*_Y_ and/or *M*_A_. *F*_I_ can spread when activity of a single *F* or *F*_I_ allele is sufficient to induce feminization; when activity is modulated by temperature, this can lead to local invasions of *F*_I_ and subsequently differentiation between populations along temperature gradients. Differentiation can also occur for *M*_Y_ and *M*_A_, with *M*_Y_ being favored in absence of *F*_I_ and *M*_A_ in presence of *F*_I_. This occurs when *M*_Y_ is associated with certain fitness effects such due to linkage with sexually antagonistic variants or recessive deleterious mutations. Altogether, we show that this can lead to the coexistence of multiple gradients in SD genes as found in, e.g., the housefly *M. domestica*.

Our model can be amended to other SD systems than the *M. domestica* system on which it is based, provided that they have a basic GSD framework influenced by an environmental effect (Table 1). Environmental effects on genetic sex determination systems are being reported in an increasing number of species (Edmands, 2021; Holleley et al., 2015). Although temperature-dependent effects are well-documented, other environmental effects may also influence SD in certain systems such as hormonal imbalance in fish due to pollution (Devlin & Nagahama, 2002). Other components and assumptions of the model that are based on the housefly system may be represented differently in other species but with similar effects. For example, the impact of *M*_A_ evolution is not due to a specific mechanism of mutation, but more generally by causing a male-biased sex ratio, thereby promoting the invasion of *F*_I_. Male-biased sex ratios occur due to *M*_A_ overrepresentation in the gene pool via its de novo evolution, but the same effect can be achieved by translocation of a Y-chromosomal male-determining gene or via association with meiotic drivers (Green, 1980; Kozielska et al., 2010). Similarly, although *F*_I_ is favored due to sex ratio selection in our model, other selective processes such as sexually antagonistic selection (van Doorn & Kirkpatrick, 2010) as well as neutral processes such as genetic drift (Saunders et al., 2018; Veller et al., 2017) can also drive transitions from male to female heterogamety. Inversely, the spread of *F*_I_ in our model may be impeded by selection against intersexes, but other factors may also limit its spread. For example, when *F*_I_ is linked to a gene experiencing intralocus sexual conflict in one environment but not another (Plesnar-Bielak & Łukasiewicz, 2021; Schenkel et al., 2018), it may be favored in the first environment due to sex-specific benefits to females; if such benefits are absent in the second type, and instead it is similarly costly to males and females alike, this would restrict the spread of *F*_I_ to the first environment.

Additionally, we see that in absence of an association between *M*_Y_ and fitness effects, *M*_A_ replaces *M*_Y_ altogether due to recurrent mutation at *M*_A_ but not *M*_Y_, yet still drives the invasion of *F*_I_, showing that our model does not strictly require a third locus. Inversely, it is likely that a more complex genetic basis generates similar evolutionary patterns, such as when various genes can evolve into a male-determining gene (Bopp, 2010). Bopp (2010) describes how a mutation that interferes with the key switch *transformer*, which regulates female development in houseflies and several other species (see also Box 1), is more likely to occur than a mutation that enhances the function of *transformer*, simply because there are many ways in which a gene’s function can be disrupted, but only a single way for it to function as intended. In our model, this mechanism may apply when *F* function can be disrupted in a variety of ways (though a similar reasoning could be applied to a gene with male determining function). In this scenario, many genes having a small chance to evolve into a male-determining gene may have the same net effect on sex ratio as a gene that is prone to evolving a masculinizing function. In this light, it will be interesting to test whether genes that have acquired sex-determining function in one species are prone to evolve a similar function in a related species, where it has no sex-determining function.

Previous work has shed light on the evolution of ESD and GSD systems, and when transitions between these two may arise (Muralidhar & Veller, 2018; Pen et al., 2010; Schwanz et al., 2020). However, our understanding of the evolution of polymorphic SD systems and the potential for environmental heterogeneity to influence this process remains limited. Our results highlight environmental effects on GSD systems, and under which conditions this can lead to within-species polymorphism in GSD systems. Alternatively, these mechanisms may also explain why in some groups GSD mechanisms may be highly divergent between closely related species, such as in the mosquitofish *Gambusia affinis* and the closely related *G. holbrooki* (Kottler et al., 2020). In such cases, different species experience different ecological conditions in their respective niches, among which environmental factors that might impinge on the SD process as described here. Between-species divergence in SD mechanisms may then occur in much the same way as occurs in our model between populations. Our results furthermore suggest that hybridization need not be an impediment to such differentiation; potentially, such diversification may even enable the evolution of reproductive isolation via Haldane’s rule (Haldane, 1922).

In conclusion, even in systems that appear to be fully GSD, the role of environmental influences on the SD processes must not be ignored as these may have played an important role in their evolution. In extension of this, changes in environment, e.g., due to global warming, may impose yet unforeseen selective pressures on species with GSD systems.

## Supplementary Material

qrad011_suppl_Supplementary_Material

## Data Availability

Source code for the agent-based model and data analysis scripts are made freely available via GitHub (https://github.com/MartijnSchenkel/Environmental_GSD_evolution).

## References

[CIT0001] Adhikari, K., Son, J. H., Rensink, A. H., Jaweria, J., Bopp, D., & Beukeboom, L. W., & Meisel, R. P. (2021). Temperature-dependent effects of house fly proto-Y chromosomes on gene expression could be responsible for fitness differences that maintain polygenic sex determination. Molecular Ecology, 30, 5704–5720. 34449942 10.1111/mec.16148

[CIT0002] Alho, J. S., Matsuba, C., & Merilä, J. (2010). Sex reversal and primary sex ratios in the common frog (*Rana temporaria*). Molecular Ecology, 19(9), 1763–1773. 10.1111/j.1365-294X.2010.04607.x 20345673

[CIT0003] Bachtrog, D. (2013). Y-chromosome evolution: Emerging insights into processes of Y-chromosome degeneration. Nature Reviews Genetics, 14(2), 113–124. 10.1038/nrg3366 PMC4120474 23329112

[CIT0004] Bachtrog, D., Mank, J. E., Peichel, C. L., Kirkpatrick, M., Otto, S. P., Ashman, T. L., Hahn, M. W., Kitano, J., Mayrose, I., Ming, R., Perrin, N., Ross, L., Valenzuela, N., & Vamosi, J. C.; Tree of Sex Consortium. (2014). Sex determination: Why so many ways of doing it?. PLoS Biology, 12(7), e1001899. 10.1371/journal.pbio.1001899 24983465 PMC4077654

[CIT0005] Berta, P., Hawkins, J. R., Sinclair, A. H., Taylor, A., Griffiths, B. L., Goodfellow, P. N., & Fellous, M. (1990). Genetic evidence equating *SRY* and the testis-determining factor. Nature, 348(6300), 448–450. 10.1038/348448A0 2247149

[CIT0006] Beukeboom, L. W., & Perrin, N. (2014). The evolution of sex determination (1st ed). Oxford University Press.

[CIT0007] Bopp, D. (2010). About females and males: Continuity and discontinuity in flies. Journal of Genetics, 89(3), 315–323. 10.1007/s12041-010-0043-9 20876998

[CIT0008] Bull, J. J., & Charnov, E. L. (1977). Changes in the heterogametic mechanism of sex determination. Heredity (Edinb), 39(1), 1–14. 10.1038/hdy.1977.38 268319

[CIT0009] Castiglia, R., Bezerra, A. M. R., Flores-Villela, O., Annesi, F., Muñoz, A., & Gornung, E. (2013). Comparative cytogenetics of two species of ground skinks: *Scincella assata* and *S. cherriei* (Squamata: Scincidae: Lygosominae) from Chiapas, Mexico. Acta Herpetol, 8, 69–73.

[CIT0010] Delclos, P. J., Adhikari, K., Hassan, O., Cambric, J. E., Matuk, A. G., Presley, R. I., Tran, J., Sriskantharajah, V., & Meisel, R. P. (2021). Thermal tolerance and preference are both consistent with the clinal distribution of house fly proto-Y chromosomes. Evolution Letters, 5(5), 495–506. 10.1002/evl3.248 34621536 PMC8484723

[CIT0011] Devlin, R. H., & Nagahama, Y. (2002). Sex determination and sex differentiation in fish: An overview of genetic, physiological, and environmental influences. Aquaculture, 208(3-4), 191–364. 10.1016/s0044-8486(02)00057-1

[CIT0012] van Doorn, G. S. (2014). Evolutionary transitions between sex-determining mechanisms: A review of theory. Sexual Development, 8(1–3), 7–19. 10.1159/000357023 24335102

[CIT0013] van Doorn, G. S., & Kirkpatrick, M. (2010). Transitions between male and female heterogamety caused by sex-antagonistic selection. Genetics, 186(2), 629–645. 10.1534/genetics.110.118596 20628036 PMC2954476

[CIT0014] Doums, C., Bremond, P., Delay, B., & Jarne, P. (1996). The genetical and environmental determination of phally polymorphism in the freshwater snail *Bulinus truncatus*. Genetics, 142(1), 217–225. 10.1093/genetics/142.1.217 8770599 PMC1206950

[CIT0015] Dübendorfer, A., Hediger, M., Burghardt, G., & Bopp, D. (2002). *Musca domestica*, a window on the evolution of sex-determining mechanisms in insects. International Journal of Developmental Biology, 46(1), 75–79. 11902690

[CIT0016] Dufresnes, C., Litvinchuk, S. N., Rozenblut-Kościsty, B., Rodrigues, N., Perrin, N., Crochet, P. A., & Jeffries, D. L. (2020). Hybridization and introgression between toads with different sex chromosome systems. Evolution Letters, 4(5), 444–456. 10.1002/evl3.191 33014420 PMC7523563

[CIT0017] Edmands, S. (2021). Sex ratios in a warming world: Thermal effects on sex-biased survival, sex determination, and sex reversal. Journal of Heredity, 112(2), 155–164. 10.1093/jhered/esab006 33585893

[CIT0018] Ezaz, T., Sarre, S. D., O’Meally, D., Marshall Graves, J. A., & Georges, A. (2009). Sex chromosome evolution in lizards: Independent origins and rapid transitions. Cytogenetic and Genome Research, 127(2-4), 249–260. 10.1159/000300507 20332599

[CIT0019] Feldmeyer, B. (2009). The effect of temperature on sex determination. University of Groningen.

[CIT0020] Feldmeyer, B., Kozielska, M., Weissing, F. J., Beukeboom, L. W., & Pen, I. (2008). Climatic variation and the geographical distribution of sex determination mechanisms in the housefly. Evolutionary Ecology Research, 10, 797–809.

[CIT0021] Gamble, T. (2010). A review of sex determining mechanisms in geckos (Gekkota: Squamata). Sexual Development, 4(1–2), 88–103. 10.1159/000289578 20234154 PMC2855288

[CIT0022] Gamble, T., Coryell, J., Ezaz, T., Lynch, J., Scantlebury, D. P., & Zarkower, D. (2015). Restriction site-associated DNA sequencing (RAD-seq) reveals an extraordinary number of transitions among gecko sex-determining systems. Molecular Biology and Evolution, 32(5), 1296–1309. 10.1093/molbev/msv023 25657328

[CIT0023] Goodfellow, P. N., & Lovell-Badge, R. (1993). *SRY* and sex determination in mammals. Annual Review of Genetics, 27, 71–92. 10.1146/annurev.ge.27.120193.000443 8122913

[CIT0024] Green, M. M. (1980). Transposable elements in *Drosophila* and other Diptera. Annual Review of Genetics, 14, 109–120. 10.1146/annurev.ge.14.120180.000545 6260015

[CIT0025] Haldane, J. B. S. (1922). Sex ratio and unisexual sterility in hybrid animals. Journal of Genetics, 12(2), 101–109. 10.1007/bf02983075

[CIT0026] Hamm, R. L., Meisel, R. P., & Scott, J. G. (2015). The evolving puzzle of autosomal versus Y-linked male determination in *Musca domestica*. G3: Genes, Genomes, Genetics, 5, 371–384. 10.1534/g3.114.014795 PMC4349091 25552607

[CIT0027] Hediger, M., Henggeler, C., Meier, N., Perez, R., Saccone, G., & Bopp, D. (2010). Molecular characterization of the key switch *F* provides a basis for understanding the rapid divergence of the sex-determining pathway in the housefly. Genetics, 184(1), 155–170. 10.1534/genetics.109.109249 19841093 PMC2815913

[CIT0028] Herpin, A., Adolfi, M. C., Nicol, B., Hinzmann, M., Schmidt, C., Klughammer, J., Engel, M., Tanaka, M., Guiguen, Y., & Schartl, M. (2013). Divergent expression regulation of gonad development genes in medaka shows incomplete conservation of the downstream regulatory network of vertebrate sex determination. Molecular Biology and Evolution, 30(10), 2328–2346. 10.1093/molbev/mst130 23883523 PMC3888023

[CIT0029] Hodson, C. N., Hamilton, P. T., Dilworth, D., Nelson, C. J., Curtis, C. I., & Perlman, S. J. (2017). Paternal genome elimination in *Liposcelis* booklice (Insecta: Psocodea). Genetics, 206(2), 1091–1100. 10.1534/genetics.117.199786 28292917 PMC5499165

[CIT0030] Holleley, C. E., O’Meally, D., Sarre, S. D., Marshall Graves, J. A., Ezaz, T., Matsubara, K., Azad, B., Zhang, X., & Georges, A. (2015). Sex reversal triggers the rapid transition from genetic to temperature-dependent sex. Nature, 523(7558), 79–82. 10.1038/nature14574 26135451

[CIT0031] Jaenike, J. (2001). Sex chromosome meiotic drive. Annual Review of Ecology and Systematics, 32(1), 25–49. 10.1146/annurev.ecolsys.32.081501.113958

[CIT0032] Janzen, F. J., & Paukstis, G. L. (1991). Environmental sex determination in reptiles: Ecology, evolution, and experimental design. Quarterly Review of Biology, 66(2), 149–179. 10.1086/417143 1891591

[CIT0033] Keating, S. E., Blumer, M., Grismer, L. L., Lin, A., Nielsen, S. V., & Thura, M. K., Wood, P. L., Quah, E. S. H., & Gamble, T. (2021). Sex chromosome turnover in bent-toed geckos (Cyrtodactylus). Genes (Basel), 12, 1–11. 10.3390/genes12010116 PMC7832896 33477871

[CIT0034] Kopp, A. (2012). *Dmrt* genes in the development and evolution of sexual dimorphism. Trends in Genetics, 28(4), 175–184. 10.1016/j.tig.2012.02.002 22425532 PMC3350790

[CIT0035] Kottler, V. A., Feron, R., Nanda, I., Klopp, C., Du, K., Kneitz, S., Helmprobst, F., Lamatsch, D. K., Lopez-Roques, C., Lluch, J., Journot, L., Parrinello, H., Guiguen, Y., & Schartl, M. (2020). Independent origin of XY and ZW sex determination mechanisms in mosquitofish sister species. Genetics, 214(1), 193–209. 10.1534/genetics.119.302698 31704715 PMC6944411

[CIT0036] Kozielska, M., Feldmeyer, B., Pen, I., Weissing, F. J., & Beukeboom, L. W. (2008). Are autosomal sex-determining factors of the housefly (*Musca domestica*) spreading north? Genet. Res. Cambridge, 90, 157–165. 18426619 10.1017/S001667230700907X

[CIT0037] Kozielska, M., Pen, I., Beukeboom, L. W., & Weissing, F. J. (2006). Sex ratio selection and multi-factorial sex determination in the housefly: A dynamic model. Journal of Evolutionary Biology, 19(3), 879–888. 10.1111/j.1420-9101.2005.01040.x 16674584

[CIT0038] Kozielska, M., Weissing, F. J., Beukeboom, L. W., & Pen, I. (2010). Segregation distortion and the evolution of sex-determining mechanisms. Heredity (Edinb), 104(1), 100–112. 10.1038/hdy.2009.104 19672280

[CIT0039] Ma, W. -J., Rodrigues, N., Sermier, R., Brelsford, A., & Perrin, N. (2016). *Dmrt1* polymorphism covaries with sex-determination patterns in *Rana temporaria*. Ecology and Evolution, 6(15), 5107–5117. 10.1002/ece3.2209 27551369 PMC4891206

[CIT0040] Meisel, R. P., Davey, T., Hak Son, J., Gerry, A. C., Shono, T., & Scott, J. G. (2016). Is multifactorial sex determination in the house fly, *Musca domestica* L., stable over time?. Heredity (Edinb), 107, 615–625. 10.1093/jhered/esw051 27540102

[CIT0041] Meisel, R. P., Olafson, P. U., Adhikari, K., Guerrero, F. D., Konganti, K., & Benoit, J. B. (2020). Sex chromosome evolution in muscid flies. G3: Genes, Genomes, Genetics, 10, 1341–1352. 32051221 10.1534/g3.119.400923 PMC7144080

[CIT0042] Mezzasalma, M., Guarino, F. M., & Odierna, G. (2021). Lizards as model organisms of sex chromosome evolution: What we really know from a systematic distribution of available data? Genes (Basel), 12. 10.3390/genes12091341 PMC8468487 34573323

[CIT0043] Miyoshi, K., Hattori, R. S., Strüssmann, C. A., Yokota, M., & Yamamoto, Y. (2020). Phenotypic/genotypic sex mismatches and temperature-dependent sex determination in a wild population of an Old World atherinid, the cobaltcap silverside *Hypoatherina tsurugae*. Molecular Ecology, 29, 2349–2358. 32474976 10.1111/mec.15490

[CIT0044] Muralidhar, P., & Veller, C. (2018). Sexual antagonism and the instability of environmental sex determination. Nature Ecology and Evolution, 2(2), 343–351. 10.1038/s41559-017-0427-9 29335574

[CIT0045] Nigro, R. G., Campos, M. C. C., & Perondini, A. L. P. (2007). Temperature and the progeny sex-ratio in *Sciara ocellaris* (Diptera, Sciaridae). Genetics and Molecular Biology, 30, 152–158.

[CIT0046] Odierna, G., Aprea, G., Capriglione, T., Castellano, S., & Balletto, E. (2007). Cytological evidence for population-specific sex chromosome heteromorphism in Palaearctic green toads (Amphibia, Anura). Journal of Bioscience, 32(4), 763–768. 10.1007/s12038-007-0076-2 17762149

[CIT0047] Ogata, M., Lambert, M., Ezaz, T., & Miura, I. (2018). Reconstruction of female heterogamety from admixture of XX-XY and ZZ-ZW sex-chromosome systems within a frog species. Molecular Ecology, 27(20), 4078–4089. 10.1111/mec.14831 30086193

[CIT0048] Palusa, S. G., Ali, G. S., & Reddy, A. S. N. (2007). Alternative splicing of pre-mRNAs of Arabidopsis serine/arginine-rich proteins: Regulation by hormones and stresses. Plant Journal, 49(6), 1091–1107. 10.1111/j.1365-313X.2006.03020.x 17319848

[CIT0049] Pane, A., De Simone, A., Saccone, G., & Polito, C. (2005). Evolutionary conservation of *Ceratitis capitata transformer* gene function. Genetics, 171(2), 615–624. 10.1534/genetics.105.041004 15998727 PMC1456775

[CIT0050] Patawang, I., Chuaynkern, Y., Supanuam, P., Maneechot, N., Pinthong, K., & Tanomtong, A. (2018). Cytogenetics of the skinks (Reptilia, Scincidae) from Thailand; IV: Newly investigated karyotypic features of *Lygosoma quadrupes* and *Scincella melanosticta*. Caryologia, 71, 29–34.

[CIT0051] Pen, I., Uller, T., Feldmeyer, B., Harts, A., While, G. M., & Wapstra, E. (2010). Climate-driven population divergence in sex-determining systems. Nature, 468(7322), 436–438. 10.1038/nature09512 20981009

[CIT0052] Plesnar-Bielak, A., & Łukasiewicz, A. (2021). Sexual conflict in a changing environment. Biological Review, 96, 1854–1867. 10.1111/brv.12728 PMC8518779 33960630

[CIT0053] Pomiankowski, A., Nöthiger, R., & Wilkins, A. (2004). The evolution of the *Drosophila* sex-determination pathway. Genetics, 166(4), 1761–1773. 10.1534/genetics.166.4.1761 15126396 PMC1470811

[CIT0054] Rodrigues, N., Merilä, J., Patrelle, C., & Perrin, N. (2014). Geographic variation in sex-chromosome differentiation in the common frog (*Rana temporaria*). Molecular Ecology, 23(14), 3409–3418. 10.1111/mec.12829 24935195

[CIT0055] Sato, T., Endo, T., Yamahira, K., Hamaguchi, S., & Sakaizumi, M. (2005). Induction of female-to-male sex reversal by high temperature treatment in Medaka, *Oryzias latipes*. Zoological Science, 22(9), 985–988. 10.2108/zsj.22.985 16219978

[CIT0056] Saunders, P. A., Neuenschwander, S., & Perrin, N. (2018). Sex chromosome turnovers and genetic drift: A simulation study. Journal of Evolutionary Biology, 31(9), 1413–1419. 10.1111/jeb.13336 29923246

[CIT0057] Schenkel, M. A., & Beukeboom, L. W. (2016). Sex chromosome evolution: Birth, maturation, decay, and rebirth. In: R. Kliman (ed.) Encyclopedia of evolutionary biology (pp. 72–80). London, UK: Academic Press.

[CIT0058] Schenkel, M. A., Pen, I., Beukeboom, L. W., & Billeter, J. -C. (2018). Making sense of intralocus and interlocus sexual conflict. Ecology and Evolution, 8(24), 13035–13050. 10.1002/ece3.4629 30619603 PMC6309128

[CIT0059] Schmid, M., Feichtinger, W., Steinlein, C., Haaf, T., Schartl, M., Visbal García, R., Manzanilla Pupo, J., & Fernández Badillo, A. (2002a). Chromosome banding in Amphibia: XXVI. Coexistence of homomorphic XY sex chromosomes and a derived Y-autosome translocation in *Eleutherodactylus maussi* (Anura, Leptodactylidae). Cytogenetic and Genome Research, 99(1-4), 330–343. 10.1159/000071612 12900583

[CIT0060] Schmid, M., Feichtinger, W., Steinlein, C., Rupprecht, A., Haaf, T., & Kaiser, H. (2002b). Chromosome banding in Amphibia: XXIII. Giant W sex chromosomes and extremely small genomes in *Eleutherodactylus euphronides* and *Eleutherodactylus shrevei* (Anura, Leptodactylidae). Cytogenetic and Genome Research, 97(1-2), 81–94. 10.1159/000064055 12438744

[CIT0061] Schmidt, R., Hediger, M., Nöthiger, R., & Dübendorfer, A. (1997). The mutation *masculinizer* (*man*) defines a sex-determining gene with maternal and zygotic functions in *Musca domestica* L. Genetics, 145(1), 173–183. 10.1093/genetics/145.1.173 9017399 PMC1207776

[CIT0062] Schwanz, L. E., Georges, A., Holleley, C. E., & Sarre, S. D. (2020). Climate change, sex reversal and lability of sex-determining systems. Journal of Evolutionary Biology, 33(3), 270–281. 10.1111/jeb.13587 31951035

[CIT0063] Sharma, A., Heinze, S. D., Wu, Y., Kohlbrenner, T., Morilla, I., & Brunner, C., Wimmer, E. A., van de Zande, L., Robinson, M. D., Beukeboom, L. W., & Bopp, D. (2017). Male sex in houseflies is determined by *Mdmd*, a paralog of the generic splice factor gene *CWC22*. Science (80-.), 356, 642–645. 10.1126/science.aam5498 28495751

[CIT0064] Sinclair, A. H., Berta, P., Palmer, M., Hawkins, J. R., Griffiths, B. L., & Smith, J. M., Foster, J. W., Frischauf, A.-M., Lovell-Badge, R., & Goodfellow, P. N. (1990). A gene from the human sex-determining region encodes a protein with homology to a conserved DNA-binding motif. Nature, 346, 240–244. 1695712 10.1038/346240a0

[CIT0065] Son, J. H., Kohlbrenner, T., Heinze, S., Beukeboom, L. W., Bopp, D., & Meisel, R. P. (2019). Minimal effects of proto-Y chromosomes on house fly gene expression in spite of evidence that selection maintains stable polygenic sex determination. Genetics, 213(1), 313–327. 10.1534/genetics.119.302441 31315889 PMC6727804

[CIT0066] Stöck, M., Savary, R., Betto-Colliard, C., Biollay, S., Jourdan-Pineau, H., & Perrin, N. (2013). Low rates of X-Y recombination not turnovers account for homomorphic sex chromosomes in several diploid species of Palearctic green toads (*Bufo viridis* subgroup). Journal of Evolutionary Biology, 26(3), 674–682. 10.1111/jeb.12086 23316809

[CIT0067] Takehana, Y., Hamaguchi, S., & Sakaizumi, M. (2008). Different origins of ZZ/ZW sex chromosomes in closely related medaka fishes, *Oryzias javanicus* and *O. hubbsi*. Chromosome Research, 16, 801–811. 18607761 10.1007/s10577-008-1227-5

[CIT0068] Takehana, Y., Naruse, K., Hamaguchi, S., & Sakaizumi, M. (2007). Evolution of ZZ/ZW and XX/XY sex-determination systems in the closely related medaka species, *Oryzias hubbsi* and *O. dancena*. Chromosoma, 116(5), 463–470. 10.1007/s00412-007-0110-z 17882464

[CIT0069] Uller, T., & Helanterä, H. (2011). From the origin of sex-determining factors to the evolution of sex-determining systems. Quarterly Review of Biology, 86(3), 163–180. 10.1086/661118 21954700

[CIT0070] Van Dooren, T. J. M., & Leimar, O. (2003). The evolution of environmental and genetic sex determination in fluctuating environments. Evolution (N. Y), 57, 2667–2677. 14761048

[CIT0071] Veller, C., Muralidhar, P., Constable, G. W. A., & Nowak, M. A. (2017). Drift-induced selection between male and female heterogamety. Genetics, 207(2), 711–727. 10.1534/genetics.117.300151 28821587 PMC5629334

[CIT0072] Verhulst, E. C., van de Zande, L., & Beukeboom, L. W. (2010). Insect sex determination: It all evolves around *transformer*. Current Opinion in Genetics and Development, 20(4), 376–383. 10.1016/j.gde.2010.05.001 20570131

[CIT0073] Wilkins, A. S. (1995). Moving up the hierarchy: A hypothesis on the evolution of a genetic sex determination pathway. Bioessays, 17(1), 71–77. 10.1002/bies.950170113 7702596

[CIT0074] Wood, S. N. (2017). Generalized additive models: An introduction with R (2nd ed). Chapman & Hall/CRC.

[CIT0075] Zhang, X., Wagner, S., Holleley, C. E., Deakin, J. E., Matsubara, K., & Deveson, I. W., O’Meally, D., Patel, H. R., Ezaz, T., Li, Z., Wang, C., Edwards, M., Marshall Graves, J. A., & Georges, A. (2022). Sex-specific splicing of Z- and W-borne *nr5a1* alleles suggests sex determination is controlled by chromosome conformation. Proceedings of the National Academy of Sciences, 119. 10.1073/pnas.2116475119 PMC8795496 35074916

