# Supplementary material for: Divergent evolution of genetic sex determination mechanisms along environmental gradients
Source: Evol Lett. 2023 Apr 1;7(3):132–47. doi: 10.1093/evlett/qrad011 (PMC10210438; doi:10.1093/evlett/qrad011)
Supplement: qrad011_suppl_Supplementary_Material [file qrad011_suppl_supplementary_material.pdf]

Supplementary Information

Divergent evolution of genetic sex determination mechanisms along environmental gradients

Martijn A. Schenkel, Jean-Christophe Billeter, Leo W. Beukeboom & Ido Pen

Supplementary Methods

An overview of all parameters and their default values are provided in Supplementary Table 2.

Life cycle

We simulate a population consisting of individuals distributed among N demes (K individuals per deme, for a total population size NK) arranged along a linear gradient (Figure 1B); we vary the environmental cue T normalized from 0 in the first deme to 1 in the last deme. T positively affects the expression of a feminizing locus F and may thereby increase the probability of an individual developing as a female. In each deme, we generate a fixed number of K individuals upon initiation. Individuals have a diploid genome consisting of three linkage groups, of which one carries the F locus. The two remaining linkage groups can carry a male-determining M locus, whose product M degrades the F product. We designate one of these linkage groups as being the original sex chromosome pair and accordingly refer to these as XY chromosome pair; we refer to the M-locus on this chromosome pair as M_{Y}. The other linkage group is referred to as the autosomal chromosome pair, and we refer to its M-locus as M_{A}. Sex is determined by the total amount of F product available after interacting with M. We assume a male heterogametic system in which females carry two X-chromosomes that lack an active M allele, and males carry one X-chromosome and a Y-chromosome that harbors an active M_{Y} allele. All individuals initially lack active M_{A} alleles. Reproduction occurs by random mating between males and females within each deme, during which all linkage groups follow Mendelian segregation. Each allelic trait can mutate with a certain trait-specific probability. Reproduction results in a total of K juveniles within each deme, which may then disperse with a probability d to a random neighbouring deme (or $d/2$ in the first/last demes). After dispersal, all adults die and are replaced by the juveniles. For all simulations, a “burn-in” period of 20,000 generations is applied during which all demes have a $T = 0$. After this, we increase T in each deme to its final value (as determined by their position in the gradient) during a warmup period of 10,000 generations. We use a burn-in and warmup phase to ensure that the system can evolve to a stable state prior to incorporating environmental effects and to ensure selection due to environmental effects does not change abruptly. After the warmup phase, we keep conditions stable for 200,000

generations to allow the system to evolve to a new equilibrium. We then analyze the genotypes of all individuals to determine which SD systems have evolved in which demes.

Sex determination

An individual's sex is determined by the net activity z_F of the two alleles at the *F* locus, based on the initial expression level of each *F* allele minus the amount of *F* product that is degraded by *M* (see Figure 1A). Initially, each allele produces an amount ${z_F}_0$ of F product which has a sensitivity s_{F_M} to breakdown by *M*. Environmental effects on SD are included solely with regard to *F* expression, so that the gross expression z_{F_g} (prior to eventual breakdown by *M*) of an F allele is given by:

$$z_{F_\mathrm{g}} = z_{F_0}\,(1+\beta T)+\varepsilon \qquad (1)$$

Here, β refers to the rate at which *F* expression increases between $T=0$ and $T=1$. To model within-deme heterogeneity in *T* and developmental noise, we add some noise to the expression of *F* by adding a Gaussian amount ε with $\mu=0$ and $\sigma=\sigma_F$. The cumulative amount $\widehat{z_M}$ of *M* product is determined by summing up the expression levels z_M of all *M* alleles. *F* breakdown by *M* is determined per *F* allele by the expression level and sensitivity of the product, so that the net activity z_F of an *F* allele is given by:

$$z_F = \max\,(0, ({z_F}_0(1+\beta T)+\varepsilon)(1-S_{F_M}\widehat{z_M})) \qquad (2)$$

We sum up the net activity of both alleles of *F* to obtain the net activity of the *F* locus, $\widehat{z_F}$, based on which sex is determined:

$$\widehat{z_F} = \sum_{i=1}^{2} {z_F}_i \qquad (3)$$

in which i is used to indicate the maternal and paternal allele. Individuals develop into males if $\widehat{z_F} < \theta_M$ or into females if $\widehat{z_F} > \theta_F$. If $\theta_M \leq \widehat{z_\mathrm{F}} \leq \theta_F$ individuals develop into infertile intersexes. *F* expression and sensitivity, as well as *M* expression, can vary from 0 to 1.

Reproduction and mutation

Reproduction occurs by mating between females and males residing in the same deme; an individual’s probability of being sampled as a mate is proportional to its fitness relative to other same-sex individuals and depends solely on sex chromosome genotype; in absence of Y-chromosomal fitness effects mating effectively occurs between randomly-sampled males and females. Mutations can occur within each gamete, and occur independently for *F* sensitivity, *F*

expression, and *M* expression each with a trait-specific probability μ. Mutations may either result in a certain shift in trait value (regular mutations) or in a loss-of-function type mutation where the trait value is set to zero (null mutation). When mutations occur, they have a trait-specific probability μ_{null} of being a null mutation, so that the effective rate at which null mutations occur equals $\mu_x\mu_{\text{null}}$, where μ_x indicates the specific mutation rate for a trait of interest (e.g., for *F* sensitivity, we find that null mutations occur at an effective rate $\mu_{\text{S}}\mu_{\text{null}} = 0.1 \times 0.01 = 0.001$ under default parameter settings). Regular mutations result in a change in a trait value by summing the current trait value with a value sampled from normal distributions with $\mu = 0$ and standard deviations $\sigma_{F_{\text{E}}}$, $\sigma_{F_{\text{S}}}$, and $\sigma_{M_{\text{E}}}$ for *F* sensitivity, *F* expression, and *M* expression. Once a trait value equals zero through either regular or null mutations, we consider this to be a loss of function and prevent the trait from undergoing further mutation so that there is no gain of function. New (expressed) M_{A} alleles may arise *de novo* with a frequency μ_{D}. The expression level of newly-evolved M_{A} locus is sampled from a normal distribution with mean μ_{E} and standard deviation σ_{E}.

Fitness effects of the Y-chromosome

We incorporate two possible fitness effects that are associated with M_{Y}. First, we incorporate a sexually antagonistic fitness effect of the Y-chromosome with additive fitness effects $-s_{\text{a}}$ and s_{a} in females and males (see Supplementary Table 1). This results in a positive effect in males but a negative effect in females, and resembles a scenario in which the Y-chromosomal *M*-locus is tightly linked to one or more sexually antagonistic alleles. These fitness effects are effectuated during the mating phase of our model. Second, we incorporate a fully recessive viability cost of $M_{\text{Y}}/M_{\text{Y}}$ homozygosity, whereby developing YY individuals survive to the adult stage with a probability $s_{\text{YY}} \leq 1$. This resembles the effect of mutation accumulation on the Y-chromosome. For both fitness effects, we assume that M_{Y} is fully linked to the loci under selection so that no recombination occurs.

Data analysis

We categorize all *F* alleles based on their sensitivity level and their expression level, where an *F* allele is considered insensitive if its sensitivity is equal to 0 (and sensitive if it is larger than 0), and is unexpressed when its expression is less than $\theta_{\text{M}}/2$ (and expressed if otherwise). We classify all alleles that are insensitive and expressed as F_{I} alleles, and all others as *F* alleles. We categorize all *M* alleles based on their expression level; we consider an *M* allele unexpressed when its expression is lower than $(2 - \theta_{\text{F}})/2$, and expressed if its expression equals or exceeds $(2 - \theta_{\text{F}})/2$. The

threshold for F expression is based on the assumption that an expression value below $\theta_\mathrm{M}/2$ is insufficient to prevent maleness when an individual is homozygous. Similarly, an M allele may break down a small amount of F product without preventing female development. Assuming F is fully expressed (and hence a total of 2 F product is generated), breakdown by M can at most be $2-\theta_\mathrm{F}$. When both F alleles are fully sensitive, this means that an individual can be homozygous for an M that breaks down at most $(2-\theta_\mathrm{F})/2$ and still develop into a female.

Following categorization, for each deme we recorded the frequencies of F and F_I as well as unexpressed and expressed M alleles on the maternally-inherited and paternally-inherited alleles. We used the 'mgcv' package (Wood, 2017) to fit generalized additive models (GAMs) with binomial distributions on the allele frequencies at the two extremes $T=0$ and $T=1$ for each locus. For the F locus, we did so on the allele frequency of the F_I allele on the maternally-inherited copy and for both M_Y and M_A we fit GAMs on the frequency of expressed alleles on the paternally-inherited copies. In each GAM, we included a full tensor product smooth between the parameters of interest.

To assess whether a polymorphic system resembling that found in natural housefly populations had evolved, we determined for each simulation whether (1) F_I was the minor allele at $T=0$ and the major allele at $T=1$; (2) M_Y was the major allele at $T=0$ and the minor allele at $T=1$; and (3) M_A was the minor allele at $T=0$ and the major allele at $T=1$. Minor (major) alleles are defined as having a frequency below (over) ½. Simulations that met all three criteria were considered to have evolved a housefly-like SD system. The resulting scores were used to fit a binomially-distributed GAM with a full tensor product smooth between M_A de novo rate (μ_D), sexually-antagonistic fitness effect (s_a), and YY survival rates (s_YY).

All data analysis was carried out in R (v.4.0.0; R Development Core Team, 2020) and RStudio (v.1.2.5033; RStudio Team, 2020), using the ‘cowplot’ (Wilke, 2019), ‘maps’ (Becker *et al.*, 2018), ‘mapsproj’ (McIlroy *et al.*, 2020), ‘mgcv’ (Wood, 2017), ‘tidyverse’ (Wickham *et al.*, 2019), and ‘viridis’ (Garnier, 2018) packages.

Supplementary Tables

Supplementary Table 1: Sexually antagonistic fitness effects of M_{Y}. s_a denotes the sexually antagonistic fitness effect of M_{Y} in females and males, whereas h_{F} and h_{M} denote the dominance of these fitness effects in $+/M_{\mathrm{Y}}$ heterozygotes. We assume M_{Y} to be deleterious to females but beneficial to males.

	**+/+**	**$+/M_{\mathrm{Y}}$**	**$M_{\mathrm{Y}}/M_{\mathrm{Y}}$**
**Female**	1	$1 - h_{\mathrm{F}} s_{\mathrm{a}}$	$1 - s_{\mathrm{a}}$
**Male**	1	$1 + h_{\mathrm{M}} s_{\mathrm{a}}$	$1 + s_{\mathrm{a}}$

Supplementary Table 2: Parameters in the model and their default values.

**Variable**	**Description**	**Standard value**
N	Number of demes	11
K	Population size per deme	10,000
d	Probability of dispersal by an individual after maturation	0.1
θ_{F}	Lower threshold for female development	1.2
θ_{M}	Upper threshold for male development	0.3
z_{F_0}	Baseline expression level of *F* alleles	0.7
S_{F_M}	Sensitivity of *F* allele's product to breakdown by M	0.95
$z_{M_{\mathrm{Y}}}$	Expression level of M_{Y} alleles	0.9
$z_{M_{\mathrm{A}}}$	Expression level of M_{A} alleles	0.9
s_{YY}	Survival rate of YY individuals	1
h_{F}	Dominance of M_{Y} sexually antagonistic fitness effect in females	0.5
h_{M}	Dominance of M_{Y} sexually antagonistic fitness effect in males	0.5
$\mathrm{s_a}$	Sexually antagonistic effect of M_{Y} (negative in females, positive in males)	0
μ_{null}	Proportion of mutations that are null mutations	0.01
μ_{E}	Mutation rate for *F* expression level	0.1
σ_{F_E}	Standard deviation for mutation effect for *F* expression level	0.1
$\mu_{\mathrm{E_{null}}}$	Proportion of mutations in *F* expression that are null mutations	μ_{null}
μ_{S}	Mutation rate for *F* sensitivity	0.1
$\sigma_{F_{\mathrm{S}}}$	Standard deviation for mutation effect for *F* sensitivity	0.1
$\mu_{S_{\mathrm{null}}}$	Proportion of mutations in *F* sensitivity that are null mutations	μ_{null}
μ_{M}	Mutation rate for *M* expression level	0.1
$\sigma_{M_{\mathrm{E}}}$	Standard deviation for mutation effect for *M* expression	0.1
$\mu_{M_{\mathrm{null}}}$	Proportion of mutations in *M* expression that are null mutations	0

μ_{D}	Rate at which M_{A} alleles will evolve to be expressed	NA (for values used see text)
β	Coefficient for F overexpression relative to temperature T	0
$\mu_{\mathrm{M_E}}$	Mean expression level for M_{A} when evolving *de novo*	0.9
σ_{E}	Standard deviation for M_{A} expression level when evolving *de novo*	0.05
σ_{F}	Standard deviation for noise in F expression level	0.05

Supplementary Table 3: Sex determination systems evolved under different levels of *F* activity relative to the maleness and femaleness thresholds θ_M and θ_F. Shown are the proportion of simulations in that category (total number of simulations per category are listed in the first column) in which a certain combination of female and male genotypes for *F* and *M* were found to be the most prevalent genotype. For *F* genotypes, *F* alleles are indicated by *F* (expressed and sensitive), F_I (expressed and insensitive), and F_0 (unexpressed). For *M*, we indicate the number of expressed *M* alleles in females and males. Dashes indicate that the system has not been observed under those particular conditions. We performed 10,000 independent simulations under different conditions, of which 9,927 terminated successfully (e.g., did not end with population extinction) and could be categorized.

**Relative *F* activity**	***F* genotypes (female - male)**	**Number of expressed *M* alleles (M_Y and M_A) (female - male)**						
		**0 - 0**	**0 - 1**	**1 - 1**	**2-1**	**2 - 2**	**2 - 3**	**3 - 3**
$z_F < \theta_M < \theta_F$ Total: 4374 simulations	$F/F - F/F$	-	**0.256**	-	-	-	-	-
	$F/F - F/F_0$	**0.003**	-	-	-	-	-	-
	$F_I/F_I - F_I/F_0$	**0.74**	-	**0.001**	-	-	-	-
	$F_I/F_0 - F_0/F_0$	-	-	-	-	-	-	-
$\theta_M < z_F < \theta_F$ Total: 4396 simulations	$F/F - F/F$	-	**1**	-	-	-	-	-
	$F/F - F/F_0$	-	-	-	-	-	-	-
	$F_I/F_I - F_I/F_0$	-	-	-	-	-	-	-
	$F_I/F_0 - F_0/F_0$	-	-	-	-	-	-	-
$\theta_M < \theta_F < z_F$ Total: 1157 simulations	$F/F - F/F$	-	**0.211**	-	-	-	-	-
	$F/F - F/F_0$	-	-	-	-	-	-	-
	$F_I/F_I - F_I/F_0$	-	-	-	-	-	-	-
	$F_I/F_0 - F_0/F_0$	**0.660**	-	**0.031**	**0.001**	**0.064**	**0.001**	**0.033**

Supplementary Figures

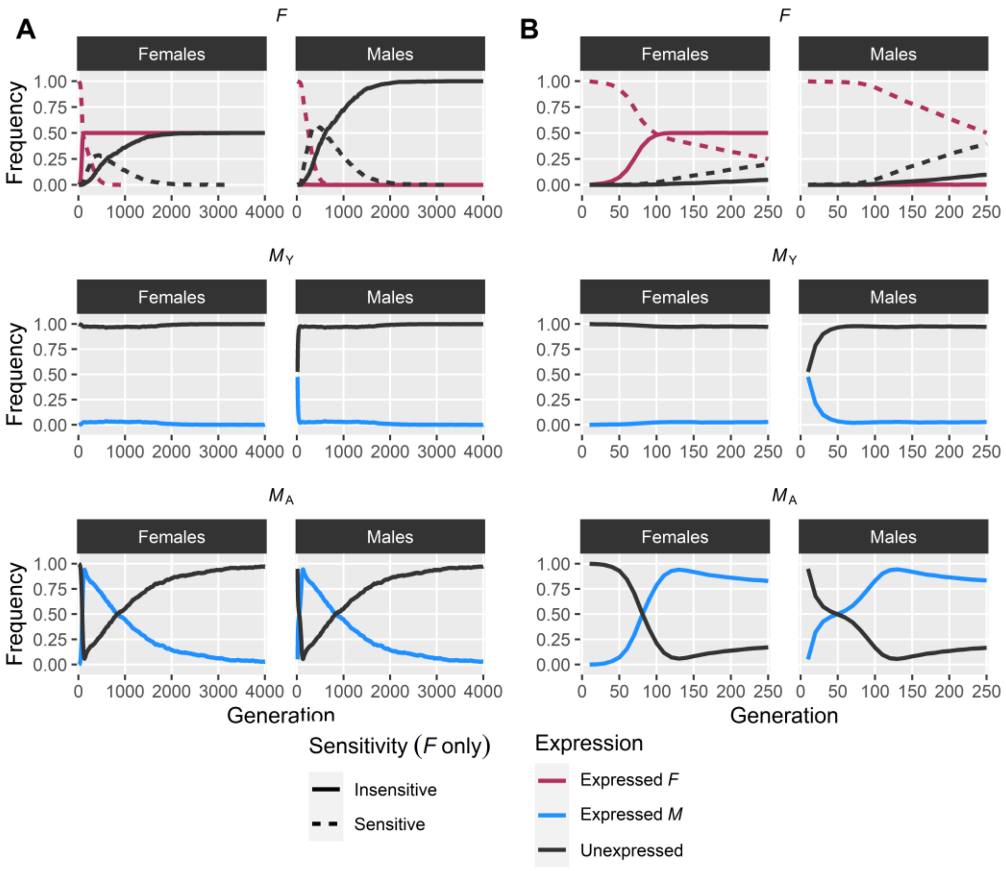

Supplementary Figure 1: Evolutionary dynamics of sex determination genes. The spread of an insensitive F allele enables the fixation of M_A in both sexes, followed by the gradual accumulation of unexpressed F and loss of expressed M_A. (A) Evolutionary dynamics over 5,000 generations. (B) Detail of the initial 250 generations shown in (A). Parameter values: $\mu_D = 0.027$; $\theta_M = 0.399$; $\theta_F = 0.789$.

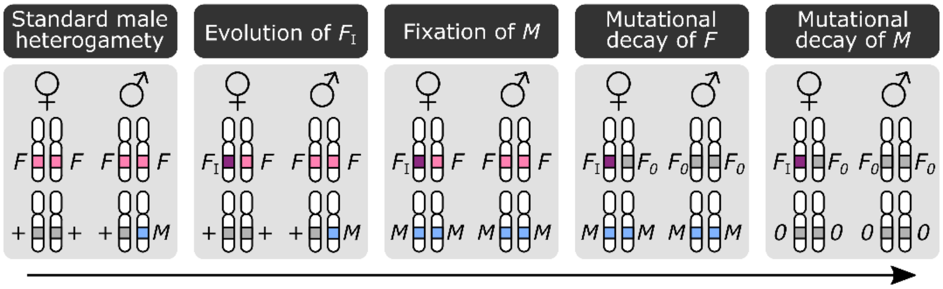

Supplementary Figure 2: Genetic decay of *F* and *M* following the evolution of an insensitive feminizing F_I allele. Starting from a standard male heterogametic system, a feminizing F_I allele can invade resulting in a transition to a female heterogamety system. For this to occur, an *M* allele must be fixed in both sexes. Fixation of *M* ensures the regularly-sensitive *F* product is always degraded and hence these alleles confer no function leaving them susceptible to genetic decay by mutation accumulation. This can eventually lead to loss of functional *F* in favor of non-expressed *F* alleles (denoted F_0). Loss of *F* obviates the need for *M* to be maintained as no regular F product is generated that must be broken down to ensure maleness in non-F_I-bearing individuals (genetic males). Similar to *F* previously, *M* functionality is no longer necessary and mutations may accumulate by which *M* becomes unexpressed (denoted 0). Note that F_I is depicted here as a dominant feminizing allele (females F_I/F, males F/F) but similar scenarios apply for a recessive feminizing F_I allele (females F_I/F_I, males F_I/F). The only difference is that the *F* allele that is susceptible to decay is now only found in males in a heterozygous state rather than in heterozygous females and homozygous males; decay of *F* and *M* can occur according to the same principles as when F_I is a dominant feminizing allele.

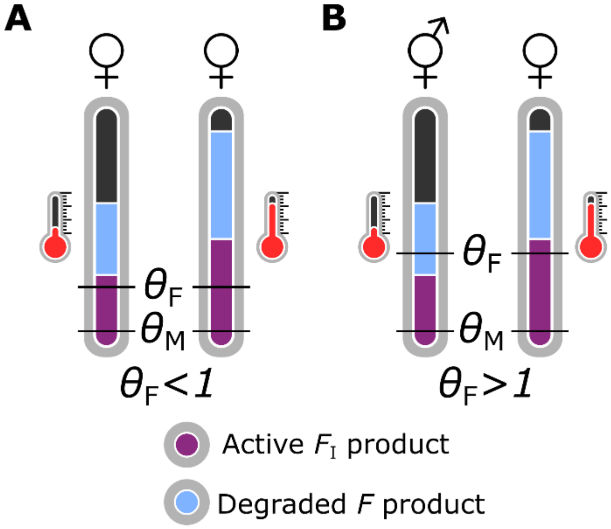

Supplementary Figure 3: Constraints on the spread of a dominant feminizing F_I allele. (A) When $\theta_F < 1$, F_I can invade in the entire population as a single *F* allele generates sufficient product to induce female development. (B) When $\theta_F > 1$, F_I cannot spread in absence of temperature-dependent overexpression as it does not generate sufficient product to induce femaleness, and instead intersexual development is induced in F_I-bearing individuals. At high temperatures, *F* overexpression enables a single *F* allele to be sufficient for feminization, allowing for F_I to spread. In all cases, we assume a F_I/*F; M/M* genotype so that regular *F* product is degraded.

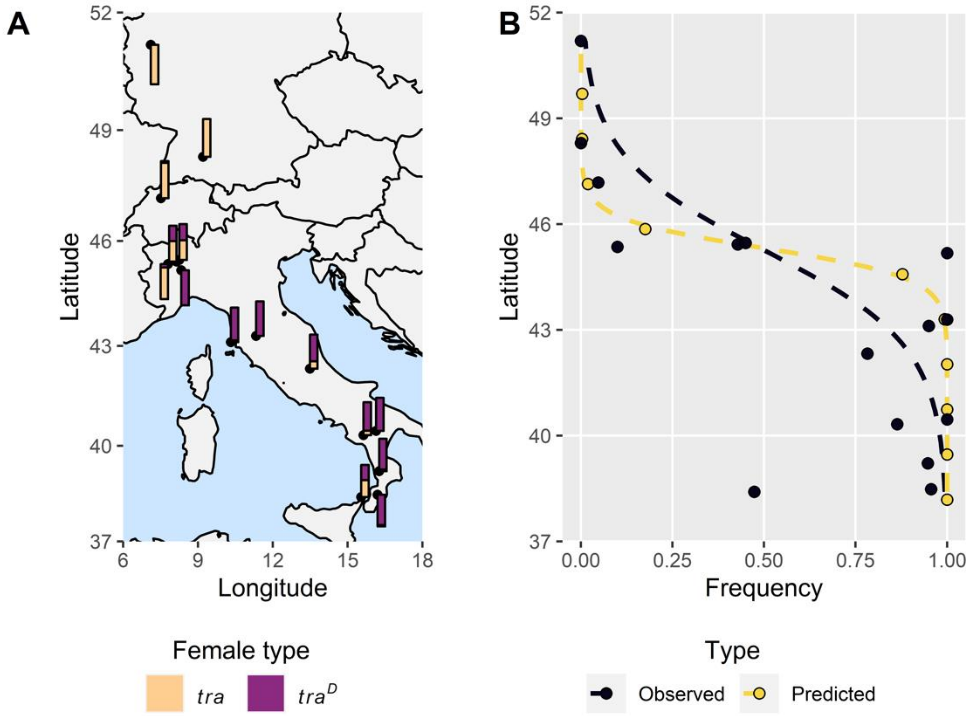

Supplementary Figure 4: Model predictions vs. the observed latitudinal frequency gradient of a female-determining gene in the housefly *Musca domestica*. (A) The frequency of *tra/tra* (light orange) and *tra*^{D}/*tra* (purple) females in Europe. Adapted from (Kozielska *et al.*, 2008). (B) Observed (black) and predicted (yellow) frequencies of *tra*^{D}-bearing females (deme position adjusted to match observed latitude range). Dashed lines indicate fitted binomial GLMs for allele frequency with latitude as the sole predictor variable. Parameter values used: $\beta = 0.5$; $\theta_{\mathrm{F}} = 1.15$; $\mu_{\mathrm{D}} = 0.005$; $s_a = 0.05$; $s_{YY} = 0.9$; $d = 0.1$.
